# Differential Hepatic Gene Expression Profile of Male Fathead Minnows Exposed to Daily Varying Dose of Environmental Contaminants Individually and in Mixture

**DOI:** 10.3389/fendo.2018.00749

**Published:** 2018-12-10

**Authors:** Ava Zare, Darren Henry, Gordon Chua, Paul Gordon, Hamid R. Habibi

**Affiliations:** ^1^Department of Biological Sciences, University of Calgary, Calgary, AB, Canada; ^2^Cumming School of Medicine, Health Sciences Centre, University of Calgary, Calgary, AB, Canada

**Keywords:** environmental contaminants, biomarker, microarray, fathead minnow, immune system, lipid metabolism

## Abstract

Environmental contaminants are known to impair reproduction, metabolism and development in wild life and humans. To investigate the mechanisms underlying adverse effects of contaminants, fathead minnows were exposed to a number of endocrine disruptive chemicals (EDCs) including Nonylphenol (NP), bisphenol-A (BPA), Di(2-ethylhexyl) phthalate (DEHP), and a mixture of the three chemicals for 21 days, followed by determination of the liver transcriptome by expression microarrays. Pathway analysis revealed a distinct mode of action for the individual chemicals and their mixture. The results showed expression changes in over 980 genes in response to exposure to these EDC contaminants individually and in mixture. Ingenuity Pathway core and toxicity analysis were used to identify the biological processes, pathways and the top regulators affected by these compounds. A number of canonical pathways were significantly altered, including cell cycle & proliferation, lipid metabolism, inflammatory, innate immune response, stress response, and drug metabolism. We identified 18 genes that were expressed in all individual and mixed treatments. Relevant candidate genes identified from expression microarray data were verified using quantitative PCR. We were also able to identify specific genes affected by NP, BPA, and DEHP individually, but were also affected by exposure to the mixture of the contaminants. Overall the results of this study provide novel information on the adverse health impact of contaminants tested based on pathway analysis of transcriptome data. Furthermore, the results identify a number of new biomarkers that can potentially be used for screening environmental contaminants.

## Highlights

- A total of 980 transcripts were differentially expressed by more than two-fold in at least one of the EDC treatments relative to the untreated control.
- More than 50% of the differentially-expressed genes in each treatment were unique for that treatment.
- 18 genes were expressed in all four EDC treatments which represent potential biomarkers for initial screening of EDC contaminants.
- Pathway analysis of transcriptome data revealed a distinct mode of action for each individual chemical and their mixture. Our results also detected some similarities in molecular pathways regulated by each treatment.
- The EDC mixture induced the integrated stress response to a greater extent than any individual treatment through inhibition of the Acute Phase Response Signaling and activation of EIF2 Signaling.

## Introduction

It is established that environmental contaminants cause adverse health effects in wildlife and humans. A number of environmental contaminants have hormone-like activity and are known as endocrine disrupting chemicals (EDCs). There is evidence that EDCs can interact with a variety of hormones and/or hormone receptors, and exert actions as agonists or antagonists. As a result, EDCs can disrupt the activity of hormones and alter normal physiological function at different levels [Reviewed in (1–3)]. Adverse effects of EDCs on the reproductive system can result from interaction with sex steroids or their receptors and other types of receptors, including aryl hydrocarbon receptor (AhR), Peroxisome proliferator-activated receptor (PPAR), liver X receptor (LXR), thyroid hormone receptors (TR), and retinoid X receptor (RXR). Thus, health impact of EDCs can result from altered gonadal development, gamete production (4–12), lower fertility, and reproductive success (13, 14), interfere with epigenetic mechanism (9, 15), altered growth (16, 17), morphology (18), metabolism (19, 20), lipid metabolism (18, 21), dysregulation of the central and peripheral endocannabinoid system (ECS) (21–23), Cardiac response and development (24, 25), DNA damage and cytotoxicity (26), neuronal development and behavior (27–29), as well as compromised immune system (3, 30) and stress performance (31). Furthermore, there is evidence that EDCs are able to increase progression of certain kinds of diseases, including obesity, diabetes, endometriosis, and hormone-dependent cancers [Reviewed in (32)].

Previous studies in our lab revealed the presence of organic contaminants downstream of treated municipal wastewater effluents and agricultural areas in Southern Alberta rivers (33–35). These studies provided evidence for female-biased sex ratio, intersex and altered gene expression in Longnose dace fish (*Rhinichthys cataractae*) in the areas containing environmental contaminants (33–35). Bisphenol A (BPA), Di(2-ethylhexyl) phthalate (DEHP), and nonylphenol ethoxylates (nonylphenol), were among the most abundant contaminants in the sites investigated. Due to their versatile uses in industry and in consumer products, these compounds are prevalent in the environment, including air soil and aquatic ecosystem, mainly through urban wastewater, industrial discharges and agricultural runoff (36–38). Reported concentration of BPA in the rivers range from below 1 to 21 μg/L. BPA can be detected as high as 72 μg/L in the municipal and paper manufacturing effluents (36, 39, 40) and in humans, ingestion due to migration to foods, beverages and infants' milk from plastic coating and bottles can reach as high as 100 μg/L (36). The NP level in surface water range from below 0.1 μg/L to 37.3 μg/L [reviewed in (41, 42)]. However, concentrations as high as 310 μg/L have been detected in surface water previously [Reviewed in (43)]. Phthalate concentrations reported range from 0.33 to 97.8 μg/L in surface water and up to 182 μg/L in sewage effluents [Reviewed in (44)]. Furthermore, a number of contaminants have hydrophobic properties and are absorbed by sediment particles. For this reason, concentrations are usually higher in sediments compared to the surface water (45, 46).

Previous studies demonstrated that exposure to low-dose (nanomolar) environmental concentrations of BPA, NP, and DEHP can alter the expression of a number of genes involved in the reproduction, gametogenesis, growth, and development (47), alter the steroid hormone levels, reduce the sperm quality (4, 5, 48), and dysregulate metabolite profile in goldfish (19). These compounds were also shown to impair the morphological development (49), disrupt hypothalamic neurogenesis and behavior in zebrafish (28).

It has been demonstrated that mixtures of contaminants with common mechanisms of action have the capacity to act in combination and exert their effects in an additive manner (50–52). The mixture effect is of particular importance when the concentration of each compound is below the threshold effect, but cumulative exposure to these chemicals can reach the effective dose to exert adverse effects (52–54). However, a number of studies have demonstrated that the effects of contaminants in mixture are synergistic rather than additive [[19. 49] for review see (52, 55–57)]. Previous studies demonstrated that mixture of BPA, NP and DEHP exert adverse effects on gene expression, metabolism and morphological development distinct from individual compounds in a non-additive manner (19, 49).

Using advanced analytical tools, an increasing number of compounds are detected in the ecosystem. However, many of these compounds are either unknown or have unknown toxicity (58). Therefore, development of effective biomonitoring tools with minimum animal would be desirable to address the risk factors associated with exposure to the contaminants. Although several techniques have been established successfully to screen for occurrence and biological effects of contaminants in the environment, their functionality is limited due to their specificity for particular classes of contaminants (59). Therefore, biomarkers, which are sensitive to various classes of contaminants, and also capable of providing information on biological outcomes of chemical exposure, would make valuable tools in ecotoxicology studies (59).

Despite considerable number of studies on environmental contaminant impacts on biological systems, and mechanisms underlying adverse effects of EDCs are less than clear [Reviewed in (60–62)]. Study of the molecular mechanisms by which environmental contaminants exert their effects will help to understand how contaminant concentration and duration of exposure, and gene regulation are linked to the adverse physiological effects at the level of organism and ecosystem. In addition, this information can potentially improve risk assessment and may even help with the regulatory processes (63). Studies using “Omics” approaches (e.g., transcriptomics, metabolomics and proteomics) can provide valuable insight into broad adverse impact of contaminants on health and ecosystems [reviewed in (63)].

Transcriptomics can provide important information on global gene expression signatures of environmental contaminants and understanding of the molecular mechanisms underlying the impact of EDCs on aquatic organisms (29, 59, 63–65). Expression microarrays can be used to identify biological pathways and common regulators targeted by contaminants individually and in mixture [Reviewed in (66)]. A number of mathematical models has been developed to predict apical parameters from transcriptome data, which has shown to be consistent with results produced from traditional methods for chemical risk assessment (67, 68). Furthermore, using gene set enrichment analysis (GSEA) based tools make it possible to use non-human model organisms to make a potential connection between human disorders and environmental conditions that lead to differentially-expressed gene sets (69).

The objective of this study was to determine the impact of BPA, NP, and DEHP exposure on the hepatic expression profile of male fathead minnow. Liver is a key organ for storage and metabolism as well as playing a major role in reproductive processes by synthesizing lipoproteins such as vitellogenin (70). Fathead minnow (*Pimephales promelas*) is a native North American species, used widely as model organism in ecotoxicology studies and regulatory testing (71). In the present study, transcriptome profiles were investigated following exposure to three EDC contaminants individually and in mixture using an expression microarray. We sought to identify the biochemical pathways targeted by these environmental contaminants, which led us to generate testable hypotheses about upstream regulators, biological processes and biomarkers. This information can be applicable in biomonitoring of the environmental contaminants as well as investigating their adverse outcomes.

## Materials and Methods

### Experimental Animals

Adult fathead minnows were purchased through Aquatic BioSystems, Inc., Colorado, US. The fish were acclimatized for 7 days prior to experimentation in the laboratory flow through glass tanks (49 L) at a constant temperature of 25°C and under a photoperiod of 16:8 L:D. Forty-five fish of mix gender per tank were selected randomly and used for each EDC treatment. Fish were fed the same amount of commercial diet of Nutrafin Max fish flakes, as recommended by the manufacturer. All protocols for maintaining and handling of fish were approved by the university animal care committee in accordance with the guidelines of the Canadian Council on Animal Care.

### Exposure to Chemicals

Bisphenol A (4,4′-(propane-2,2-diyl) diphenol) (BPA, 239658), (Di(2-ethylhexyl) phthalate) (DEHP, 80030), and 4-nonylphenol (NP, 442873), were purchased from Sigma-Aldrich (Missouri, United States). To minimize bias, fish were distributed evenly and at random among five flow-through glass aquaria, and the treatments were randomly assigned to the experimental tanks. The flow-through glass tanks were supplied with activated carbon-filtered City of Calgary water (flow rate at 300 mL/min). Fish were exposed for 21 days based on the 21-day fish endocrine assay guidelines by EPA (72) in tanks treated with 100 μg/L of individual chemicals or a mixture of the three compounds (100 μg/L of each chemical). The control group was exposed to the same concentration of the vehicle (EtOH). These EDC compounds are among the most common and abundant environmental contaminants in various parts of the world including Alberta surface water and effluents (73). In order to study the compensatory and toxicity responses of animals to EDC exposure, the concentrations used for this study were within the higher range of the reported environmental levels [(44) Reviewed in (36, 43)]. The concentration of contaminants in the environment is variable throughout the day across geographic areas depending on the distance from the wastewater effluent discharge, flow rate or volume in the receiving rivers (74). Also, as the fish swim away from the wastewater effluent discharge they would expose to variable concentration of chemicals. In the present study, we created an experimental condition to expose fish to daily steady decline in chemical concentration using a flow-through system to address the variability in the contaminants concentrations in the river to some extent. Aquarium water was changed daily with fresh water followed by adding a nominal dose of contaminants (100 μg/L). Flow rate was adjusted to gradually reduce the concentration of chemicals by 50% every 4 h. This treatment procedure ensured exposure of fish to declining concentrations of chemical throughout the day as a result of the renewal of the water. Since the chemical levels were variable during the day, we did not measure their concentrations in the water. After exposure to the chemicals for 21 days, fish were anesthetized in 250 mg/L buffered tricaine methanesulfonate (MS-222, Sigma–Aldrich, MO, United States), euthanized, weighed and liver tissue from male fish were rapidly dissected, frozen in liquid nitrogen, and stored at −80°C for future studies. Dividing the weight of the gonads by the weight of the fish and then multiplying by 100 determined the gonadosomatic index (GSI) of each fish.

### RNA Extraction

Total RNA was extracted from liver tissue using TRIzol Reagent (Invitrogen, CA, United States) according to the manufacturer's protocol (75) and treated with DNAse (Ambion, CA, United States). Total RNA was quantified verified using a NanoDropTM 1000 Spectrophotometer (Thermo Scientific, Delaware, United States) and OD 260/280 and 260/230 ratios were obtained to determine the purity of the RNA samples. A portion of RNA isolated from each treatment was saved for qPCR sufficient for 12 replicates, and the rest of extracted RNA was pooled for the microarray experiments. Total RNA samples were run on a 1% agarose gel to confirm its integrity after isolation.

### Microarray Experimental Design

We used two-channel (two-color) Agilent expression microarrays to determine the transcriptomes of male zebrafish livers exposed to BPA, DEHP, NP and a mixture of these three compounds. In this type of array compared to one-color microarray, the relative hybridization intensity of each treatment can be directly contrasted with the control sample (non-chemical treated group), which eliminates the variation due to the spot size or distribution pattern of each probe (76). Also performing the expression microarray experiments with dye swap reduces the technical variations (77). In expression microarray studies when a limited quantity of RNA is available, pooling RNA samples or preamplification of RNA samples can be utilized. However, studies have demonstrated the presence of amplification bias during cRNA synthesis (78) and also increased cross-hybridization in RNA-DNA hybridization on the expression microarrays (79). In this study, we pooled the RNA samples to generate adequate quantities to avoid amplification bias. We performed qPCR with 12 replicates to verify the expression microarray data of several relevant gene candidates.

### Microarray Protocol

mRNA from 1 to 2 mg of pooled total RNA was extracted using oligo(dT) 25-cellulose beads (NEB, Massachusette, United States) in a Poly-Prep® Chromatography Column (BioRad, CA, United States). Isolated mRNA was quantified using a NanoDropTM 1000 Spectrophotometer (Thermo Scientific, Delaware, United States). Two microgram of mRNA was reverse transcribed using Oligo (dT)23 anchored primer (Sigma-Aldrich, Missouri, United States) and SuperScript II reverse transcriptase (Invitrogen CA, United States) and aminoallyl-dUTP (Sigma-Aldrich, Missouri, USA was incorporated into the cDNA during reverse transcription. Purified cDNA samples were then labeled with Cy™3 and Cy™5 [For detailed procedure see (80)]. Fathead minnow 8 × 15K Agilent expression microarrays were used for this study, in which gene-specific probes were designed by EcoArray (FL, United States) and manufactured by Agilent (CA, United States).

Microarray hybridization was carried out using a reference design, where each cDNA sample was compared to a reference (untreated male liver control). 1 μg of labeled cDNA of each treatment was co-hybridized onto the microarray with cDNA from the untreated control following the manufacturer's recommendations (Two-color microarray hybridization protocol; Agilent, CA, United States). Dye-swap normalization was performed to remove systematic dye intensity bias from expression microarray data.

The expression microarrays were scanned with an Axon GenePix® 4200A laser scanner (Molecular Devices, CA, United States) at 5 μm resolution using wavelengths of 532 nm for the Cy™5 and 625 nm for Cy™3 dyes. Microarray data quality was assessed by manual inspection. Axon GenePix® Pro 6.0 software (Molecular Devices, CA, United States) was used to generate the raw spot intensities of the expression microarrays.

### Bioinformatics

LOWESS (Locally weighted Scatterplot Smoothing) normalization was performed on the raw expression microarray data using the R Bioconductor package LIMMA software. The average log2 ratios between the experimental channel and the control channel from the dye-swap experiments were calculated by *t*-test and corresponding *p*-values were obtained (81). Significant changes in gene expression were identified as having a *p* < 0.001. Hierarchical clustering was performed on differentially regulated transcripts (*P* < 0.001, and >± 2 fold changes) using Cluster 3.0 software (82). Java TreeView software was used for visualizing the heat map (83). In order to identify the common and unique genes that were significantly affected by chemical exposure, a Venn diagram was created using Venny 2.1 software (84).

We investigated the networks, pathways, regulators and functions associated with NP, BPA, DEHP, and mixture exposure from the expression microarray data using Ingenuity Pathway (IPA) analysis (Ingenuity Systems, CA, United States). Significantly differentially-regulated transcripts (*P* < 0.001, and >± 2 fold changes) were mapped to human homologs using the Entrez GeneID and along with their fold change information uploaded to IPA. The most significant biological functions and the top canonical pathways from the expression microarray data were identified. Based on the information from all the molecules and their relationships in the Ingenuity Knowledge Base, upstream regulators and downstream biological function and diseases associated with chemical exposure were predicted.

Z-scores and *p*-values were used to interpret the IPA results. Calculated Z-scores indicated whether it was likely that identified pathways, upstream regulators, and biological functions were activated or inhibited. *P*-values were calculated by the Fisher exact test to determine the significance of the overlaps between a set of focus genes in the treatment and a given process or pathway in IPA knowledge base. *P* < 0.05 were considered significant. Also, a comparison analysis among the four treatments was performed to determine the gene expression changes involved in certain molecular pathways of each EDC compound.

### Quantitative Real-Time PCR (qPCR)

Total RNA was reverse transcribed using Applied Biosystems High Capacity cDNA Reverse Transcription Kit (Life Technologies, CA, United States) according to the manufacturer's protocol. The cDNA was stored at −20°C prior to qPCR.

We used qPCR to validate the expression microarray results and also to verify roles of the predicted upstream regulators and biological pathways obtained by IPA on observed dysregulation of the transcriptome. A number of genes were selected based on their function, microarray expression results and IPA pathway analysis. Specific primers were designed based on the genome sequences available in GenBank or provided by EcoArray Inc. (Table 1). Primer efficiency was determined by performing qPCR on a dilution series of cDNA. Primers with 90–105% efficiency were considered for use. PCR products were confirmed by sequencing (University of Calgary Core DNA Services) and melt curve analysis performed to ensure that the primers amplified a single product. The primer sequences and annealing temperatures are listed in Table 1.

**Table 1:** List of the primers used in gene expression analysis by quantitative Real-Time PCR.

**GENE**	**Forward primer (5^**′**^-3^**′**^)**	**Reward primer (3^**′**^-5^**′**^)**	**A T**
AR	TACCCTAACGTGCCCTGTGTGA	CCGCATCAAACCTGCCATCTGT	58
Cyp19a	TTGTGCGGGTTTGGATCAATGGTG	TTCCGATACACTGCAGACCCAGTT	55
Cyp1A	TGCCCTTGAGGAGCACATCAGC	CGTCGTCGTGGCTGTAGCG	58
CYP27B1	GAGTTCTACCGCTTTGGTCTC	GCTGTTGATGGACTGGATGA	56
DMRT1	CTCCTATTACAACCTCTACC	CTGGACCGGCGACCATTTCC	57
GAPDH	TGATGCTGGTGCCCTGTATGTAGT	TGTCCTGGTTGACTCCCATCACAA	57
GSTA	CTCTGATGCTGCAGGAGTTATT	GCTGCAGGAATTTGCTGATTT	55
IFIT2	TCATCTCCAACAGAGCTTCAC	CTTGTCATCCGGCTCTTTCT	56
IGF1	AACTCCACGATCCCTACGAG	CTTCTGATGGACCTCCTTACAGG	55
INSR	GAACTATACTGTGCGGATCAGAG	CCACACGTAGGTCCTTAACATAG	57
IRF7	CGCATCCTAGACAGCATTCA	CTGGTGCTGACGAAGACTTTA	56
LRP8	ATGAAGATGCGCCAGTCACA	TGCAGGTTGGAGGGTCTTTG	57
RBP4	CGATAACTACGCCATCCACTAC	AGGGTGTCGGGAGAATATGAAG	56
RSAD2	CAGGGCAAGAAGAGCCATTTA	GTAGGTGTTGATCACGGAGTTG	55
Vtg1	GAAGTGCGCATGGTGGCTTGTATT	AGCTGCCATATCAGGAGCAGTGAT	55

qPCR reactions were carried out on a BIO-RAD CFX96 TouchTM Real- Time PCR Detection System using BIO-RAD IQTM SYBR® Supermix following the manufacturer's protocol (BIO-RAD, CA, United States). The conditions for each PCR reaction were as follows: 10 μL Supermix, 300 nM of each primer, 0.75 μL cDNA, and 8.65 μL ultrapure water (Life Technologies, CA, USA). The condition of qPCR amplification was as follows: initial denaturation at 95°C for 2 min, 40 cycles of denaturation at 95°C for 10 s and annealing/extension at a primer optimized temperature for 30 s. Reactions were carried out in triplicate to ensure consistency. Gene expression data was normalized by calculating the difference relative to the housekeeping gene. The main assumption for using a particular reference gene to normalize the data is that it should be stable with minimal variation following treatments. To maximize stability of the reporter gene, we tested the suitability of three reference genes β-actin, glyceraldehyde 3-phosphate dehydrogenase (GAPDH) and ribosomal protein subunit 18 (rps18) for stability as described previously (85). We found that β-actin and GAPDH were the least variable reference genes for normalization. To ensure the accurate normalization of the qPCR results, we used the mean of β-actin and GAPDH following the 2^−Δ*ΔCt*^ method (86). One-way ANOVA followed by *post-hoc* Tukey's test using Prism 5 statistical software (GraphPad Software, Inc., United States) was carried out on the normalized expression data to identify significant expression changes (*p* < 0.05). All values were expressed as mean ± standard error.

## Results

In the present study, fathead minnows were exposed to 100 μg/L of NP, BPA, or DEHP or a mixture of three for 21 days to investigate hepatic gene expression changes associated with toxicity induced by these compounds. Based on the results of this study no treatment related mortality was observed. Body weight, gonadosomatic index (GSI) and hepatosomatic index (HIS) also were not significantly different between the treatments.

### Gene Expression Profile in the Male FHM Liver Following NP, BPA, DEHP, and Mixture Treatment

There was a total of 980 transcripts differentially expressed by more than 2 folds (*p* < 0.0001) at least by one of these treatments compared to the control. Exposure to NP, BPA, DEHP and their mixture significantly modulated the expression of 316, 335, 381, and 247 target genes in the liver tissue relative to the control fish, respectively, (Table 2). Of these transcripts, 156, 195, 124, and 73 were up-regulated relative to control while 160, 140, 257, and 174 were down-regulated by each treatment, respectively (Table 2).

**Table 2:** Gene expression changes in the liver of male fathead minnow response to exposure to NP, BPA, DEHP and the mixture.

**Gene-expression changes**	**NP**	**BPA**	**DEHP**	**Mix**
Total regulated genes (n)	316	335	381	247
Up-regulated genes [n (%)]	156 (49.4)	195 (58.2)	124 (32.5)	73 (29.6)
Down-regulated genes [n (%)]	160 (50.6)	140 (41.8)	257 (67.5)	174 (70.4)
Uniquely regulated genes [n (%)]	158 (49.4)	177 (52.8)	212 (55.6)	140 (56.7)
Commonly regulated genes [n (%)]	18 (5.6)	18 (5.4)	18 (4.7)	18 (7.2)
Regulated genes that overlap with mixture [n (%)]	45 (14.2)	46 (13.7)	73 (19.1)	–

**Figure 1:**
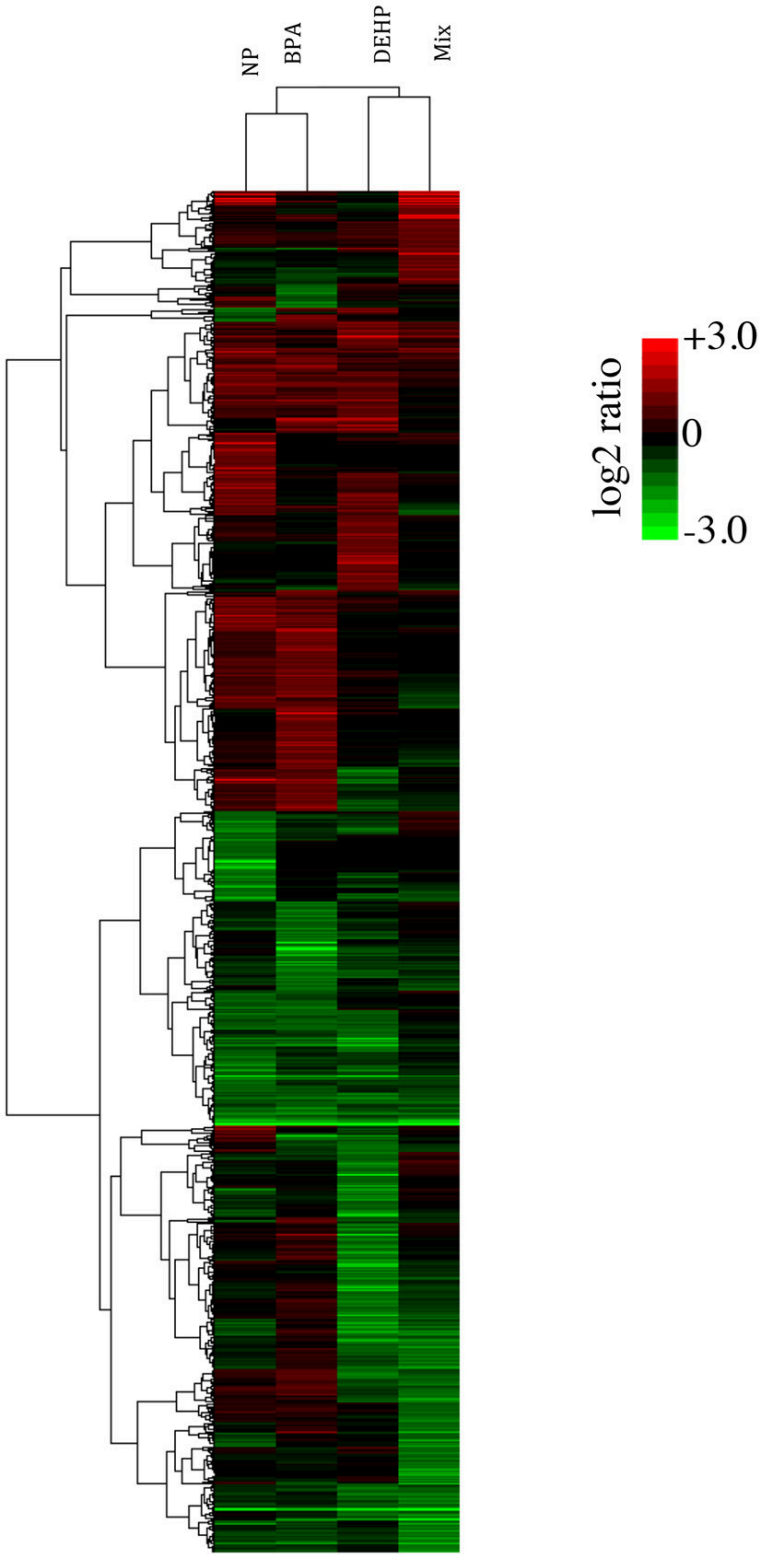
Hierarchical cluster analysis (Pearson correlation) of differentially expressed-genes in the livers of male FHMs following 21 days exposure to 100 ug/L of NP, BPA, DEHP and their tertiary mixture (*p* < 0.0001; fold change > 2). Green color represents down-regulated genes, red color represents up-regulated genes, and black color represents no change in response to EDC treatment relative to the untreated control. Color bar indicates log2 expression ratios of EDC treatment relative to untreated controls.

To compare the global pattern of transcriptional response caused by individual contaminants and the mixture, we performed hierarchical cluster analysis of the 980 transcripts identified as significantly regulated after exposure (Figure 1). Each row in the heat map represents a gene, and each column represents a chemical treatment. The cluster analysis demonstrated that NP and BPA differentially-expressed genes clustered together and DEHP clustered with the mixture group. Despite the similarities, there is a clear difference among all four treatments, suggesting that each compound induces a distinct gene expression pattern compared to the mixture.

**Figure 2:**
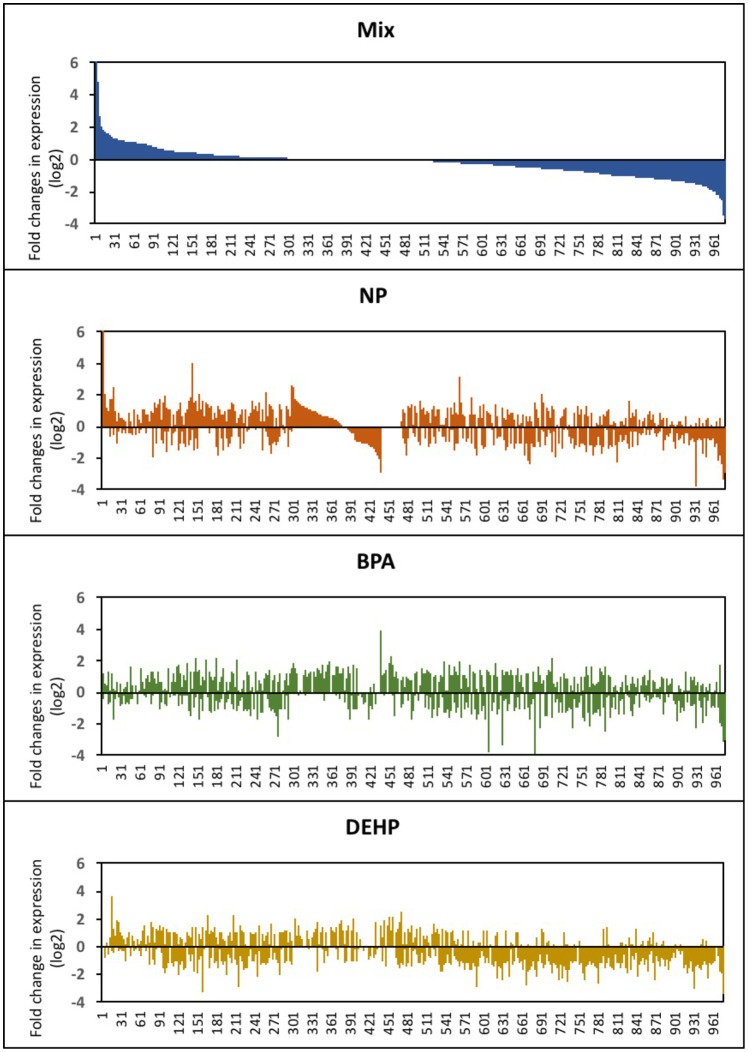
Expression profile of male fathead minnow livers following exposure to NP, BPA, DEHP and the mixture of three compounds. Differentially-expressed genes are shown as the log_2_ fold change of expression in EDC-exposed male fish compared to untreated control fish. The gene order in the horizontal axis corresponds to the differential-expressed genes from the EDC mixture is kept consistent for the other three treatments.

In Figure 2, the entire set of differentially-expressed genes was plotted in order of expression change (log2) for the mixture group and the order of the genes were kept the same for the other treatments. This graph revealed that while the mixture effects was additive for some of the transcripts, the majority of the genes was significantly altered only by the mixture. Also, a few transcripts were differentially expressed in response to individual compounds and appeared unaffected by the mixture. NP, BPA and DEHP only shared 14, 13.7, and 19.1%, respectively, of their differentially-expressed genes with the mixture (Table 2). Among the differentially-expressed genes in response to exposure to the mixture, the pattern of up and down-regulated genes was also different compared to the individual compounds.

**Figure 3:**
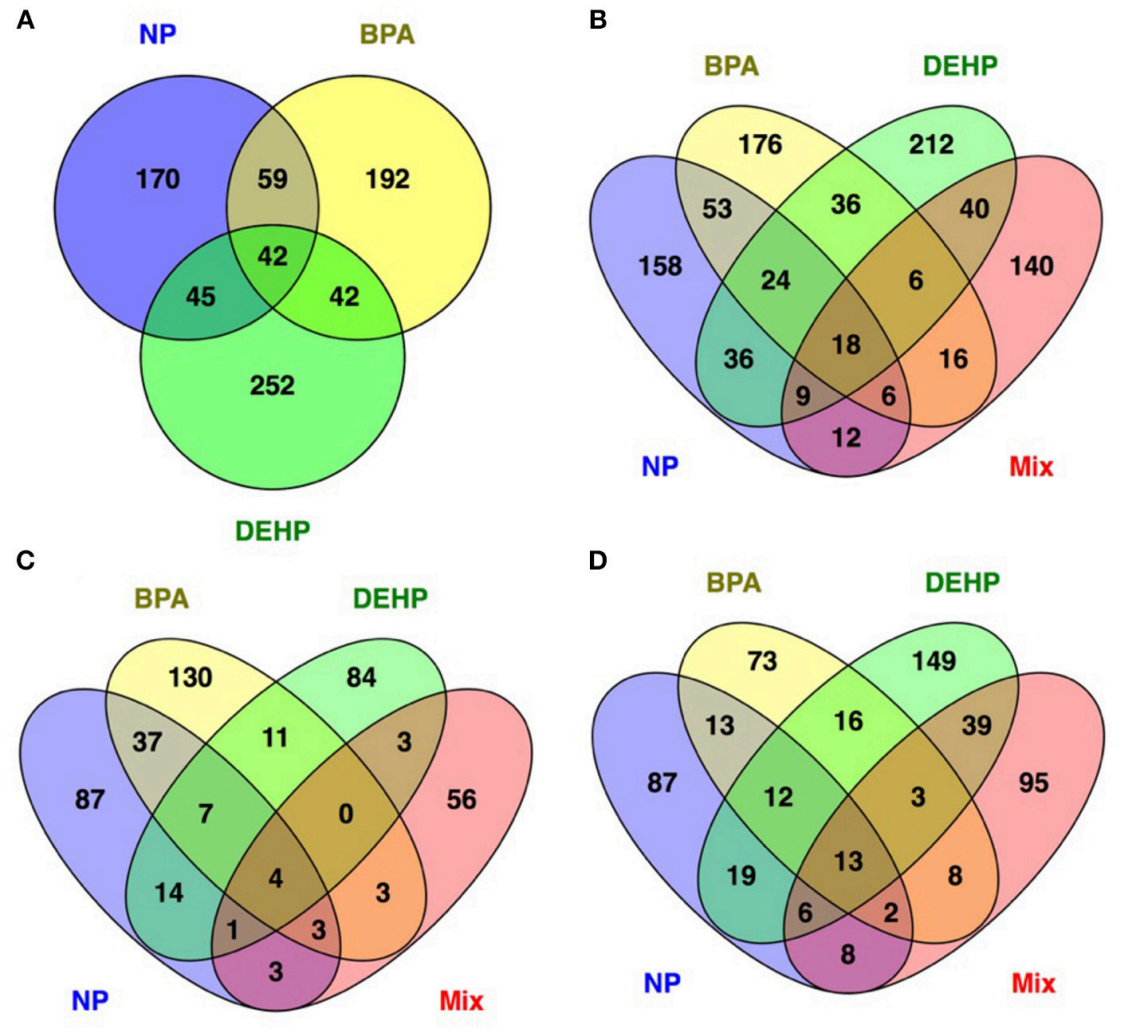
Venn diagram showing the number of unique and common differentially regulated genes in male FHM hepatic tissue affected by NP, BPA and DEHP exposure **(A)** as well as mixture **(B)**. **(C,D)** show the upregulated and down regulated genes respectively. The Venn diagrams reveal 42 genes commonly modulated by individual treatments **(A)** and 18 genes regulated by all three compounds and their mixture **(B)**. Among these 18 genes 4 of them upregulated **(C)** and 13 of them inhibited **(D)** by all four treatments.

The Venn diagram in Figure 3 showed that few differentially-expressed genes were the same among the different EDC treatments. More than 50% of all the differentially expressed genes were uniquely affected by each treatment. There were only 43 transcripts commonly modulated by all three EDC compounds (Figure 3A) and among these 43 genes, just 18 of them overlapped with the mixture group (Figure 3B). Out of these 18 genes, four genes were up-regulated (Figure 3C) and 13 genes were down-regulated (Figure 3D) in all four treatment groups, and one gene was down-regulated in NP and BPA treated groups and up-regulate in DEHP and mixture group. Examples of some of these genes commonly down-regulated by all four treatments included Interferon Regulatory Factor 7 (IRF7), Radical S-adenosyl Methionine Domain Containing 2 (RSAD2), Similar to Interferon-inducible Protein IFI56 (IFIT2), and Synaptonemal Complex Protein 1 (SYCP1). In addition, Glutathione S-transferase A1 (GSTA1) is an example of the genes up-regulated in all four treatments. The biological function and cellular processes of these genes included DNA binding, metal ion binding and homeostasis, lipid binding, transport and metabolism, innate immune response, inflammatory response, cell division and differentiation, and carbohydrate metabolism (Table S1). Commonly regulated genes by all four treatments may represent candidate biomarkers of toxicity as they have the potential to response to a variety of chemical compounds with distinct mode of actions.

### Pathway Analysis

We used IPA to investigate the biological pathways that were affected by exposure to individual compounds compared to the mixture exposure. Some of the most significantly affected pathways by mixture exposure included EIF2 Signaling, LXR/RXR Activation, PPAR/RXR Activation and Acute Phase Response Signaling pathways while PI3K/AKT Signaling and NRF2-mediated Oxidative Stress Response were mostly affected by individual chemicals (Table 3). Some examples of the identified upstream regulators included AhR, Hepatocyte growth factor (HGF), Androgen receptor (AR), Tuberous Sclerosis Complex 2 (TSC2), Apolipoprotein A-I (APOA1), Interferon Regulatory Factor 1(IRF1), and Colony Stimulating Factor 2 (CSF2) (Table 4). The molecular functions of these upstream regulators are detoxification, growth and metabolism, reproduction, lipid metabolism, and immune system (Tables S2–S5). The potential diseases and biological outcomes that are associated with the observed over represented genes included, liver inflammation, binding and storage of lipids, steroid metabolism, cancer, apoptosis, and viral infection (Tables S6).

**Table 3:** Canonical pathways and biological processes associated with significantly altered transcripts in fathead minnow liver after 21 days exposure to NP, BPA, DEHP and the mixture of the three chemicals using Ingenuity Pathways Analysis (IPA) and Fisher exact test (*P* < 0.05).

**Canonical pathway**	**Z-Score**			
	**NP**	**BPA**	**DEHP**	**Mix**
Acute phase response signaling	1.807	1.604	−1.807	−1.604
PI3K/AKT Signaling	−2.646	−1.342	−2.646	–
PPAR/RXR Activation	1.134	−1.633	2.121	1.633
Activation of IRF by Cytosolic Pattern Recognition Receptors	−1.342	−1.342	−2.236	−1
LXR/RXR Activation	1.706	−0.943	−1.225	−1.877
EIF2 Signaling	0.277	−1.508	1.387	2.496
IGF-1 Signaling	1.633	1.633	0.816	1.342
NRF2-mediated Oxidative Stress Response	−1.342	0.447	−2.236	1.342
Production of Nitric Oxide and Reactive Oxygen Species in Macrophages	0.535	0.302	−2.324	−1.387
Extrinsic Prothrombin Activation Pathway	0.816	−0.816	−1.342	−1.342
Prolactin Signaling	1.134	1.134	0.378	0.816
Growth Hormone Signaling	1.89	–	1.134	0.378
JAK/Stat Signaling	0.33	1.41	−0.33	1.13
ATM Signaling	2.0	–	1.0	–

*A positive or negative Z-score indicates activation or inhibition of the pathway*.

**Table 4:** Predicted master regulators that might be associated with observed dysregulation in the male fathead minnow liver following exposure to NP, BPA, DEHP and the mixture of three compounds for 21 days.

**Upstream regulators**	**Z-Score**				**Molecule function**
	**NP**	**BPA**	**DEHP**	**Mix**	
PXR ligandRXR	1.648	2.407	0.713	0.337	Detoxification	Detoxification
AHR	2.832	2.147	1.201	0.858	Adaptive and toxic response
RXRA	1.047	2.231	−0.929	−0.01	Growth and development	Growth and metabolism
HGF	−1.424	−2.056	−3.094	−3.235	Hepatocyte growth factor, regulates cell growth
Growth hormone	−0.295	−0.473	−2.173	−2.682	Stimulates growth, cell reproduction
GHR	1.673	−0.447	2.4	0.946	Stimulates growth, cell reproduction
PPARG	2.015	3.274	−0.929	−0.468	Metabolism and lipid uptake
AR	−1.378	−0.684	−2.44	−1.459	Androgen receptor	Reproduction
FIGLA	1.534	2.396	1.534	1.534	Involved in folliculogenesis
ER	1.691	0.228	0.036	−0.508	Nuclear receptor
FSH	−0.041	−0.438	−1.616	−1.202	Gonadotropin hormone
APOA1	–	–	1.94	−0.11	Apolipoprotein A-I	Lipid metabolism
APOA4	−1.29	−1.97	−0.21	−1.96	Apolipoprotein A-4 (equivalent to Vtg in oviparous)
LDL	0.31	1.54	−1.79	−0.45	Low-density lipoprotein
LDLR	−0.38	–	1.36	–	Low-density lipoprotein receptor
ERBB2	−2.138	−2.494	−1.464	−2.11	Tyrosine kinases receptor, involved in breast cancer
TSC2	2.219	2.219	2.219	−0.555	Involved in Tuberous sclerosis
SASH1	−2.236	−2.236	−2.236	−2.236	Down-regulates in breast cancer cells
STAT1	−1.29	−3.025	−1.721	−1.671	Cellular immunity, proliferation, apoptosis
SP1	1.462	2.292	0.366	−0.623	Including cell differentiation, cell growth, apoptosis, immune responses
DOCK8	−2.236	−2.236	−2.236	−2.236	Intracellular signaling
IRF7	−2.506	−2.506	−2.913	−2.913	Immune respons	Immune response
CSF2	−2.182	−2.956	−2.525	−2.543	Immune response
IL27	−1.725	−1.368	−2.439	−1.932	Immune response
IFI16	−1.312	−1.373	−1.679	−1.129	Innate immune response
IL1B	−1.447	−1.377	−1.023	−2.961	Immune response
IL3	−2.068	−1.385	−2.068	−0.888	Immune response
IFNAR1	−1.709	−2.432	−2.432	−2.432	Immune response, inhibits viral infection
IRF1	−2.006	−3.243	−2.293	−2.248	Immune response
IFNA2	−1.621	−2.283	−2.314	−3.144	Immune response, inhibits viral infection
STAT	−0.708	−2.398	−0.145	−2.398	Cellular immunity, proliferation, apoptosis
mir-15	1.741	2.207	1.741	−0.958	MicroRNA precursor, immune response
Rosiglitazone	0.511	2.418	−2.998	−2.062	Insulin sensitizer, by binding to the PPAR
Isobutylmethylxanthine	−0.73	2.376	−2.905	−3.132	Reduces inflammation and innate immunity
Roscovitine	1.546	2.216	1.546	−0.492	Alter the growth phase or state within the cell cycle	Chemicals/drugs
Cadmium	2.88	1.767	−1.334	−1.182	
Bisphenol A	1.009	1.367	−0.174	−0.543	
Dihydrotestosterone	0.859	1.051	−2.135	−1.906	Sex steroid
4-hydroxytamoxifen	2.578	0.133	1.165	0.209	Selective estrogen receptor modulator

*These regulators were predicted based on the significantly overrepresented genes in the dataset using Ingenuity Pathways Analysis (IPA) and Fisher exact test (P < 0.05). A positive or negative Z-score indicates activation or inhibition of the pathway*.

### Quantitative Real-Time PCR

We used qPCR to validate the expression microarray results and also confirm some of the identified biological pathways and predicted upstream regulators that were hypothesized to be affected by these treatments from the pathway analysis. We selected a few candidate genes for qPCR confirmation based on their function, microarray expression results, and IPA pathway analysis results, as listed below.

Glutathione S-transferase A1 (GSTA1), Interferon Regulatory Factor 7 (IRF7), Radical S-adenosyl Methionine Domain Containing 2 (RSAD2) and Similar to Interferon-inducible Protein IFI56 (IFIT2) were selected because they were significantly regulated by all the treatments and also involved in detoxification and immune response.

Vitellogenin (Vtg), which is a widely used biomarker of exposure to estrogenic compounds, is an Apolipoprotein and involved in the transport of lipids. Our expression microarray results also indicated that many genes involved in lipid metabolism and transport were regulated by the EDC exposure. Hence, we selected some regulated genes within this pathway such as Low Density Lipoprotein Receptor-Related Protein 8 (LRP8) and Retinol-binding protein 4 (RBP4) that were detected in the expression microarray data, as well as Low Density Lipoprotein Receptor (LDLR) which was not included in the set of microarray probes.

In order to confirm the predicted regulatory networks and biological functions that were disrupted by EDC exposure, we also quantified the expression levels of some genes involved in AhR, AR and IGF signaling as well as the PPAR/RXR activation pathway. These genes included the Insulin receptor (INSR) and cytochrome p450 27B1 (CYP27B), which were included in the expression microarray data, and cytochrome p450 1A1 (CYP1A1), insulin like growth factor1 (IGF1), gonad aromatase (CYP19a1), androgen receptor (AR), Doublesex and mab-3 related transcription factor1 (DMRT1) and LDLR (in testis) were also selected, but were not included in the set of microarray probes.

### Vitellogenin and Lipid Metabolism

**Figure 4:**
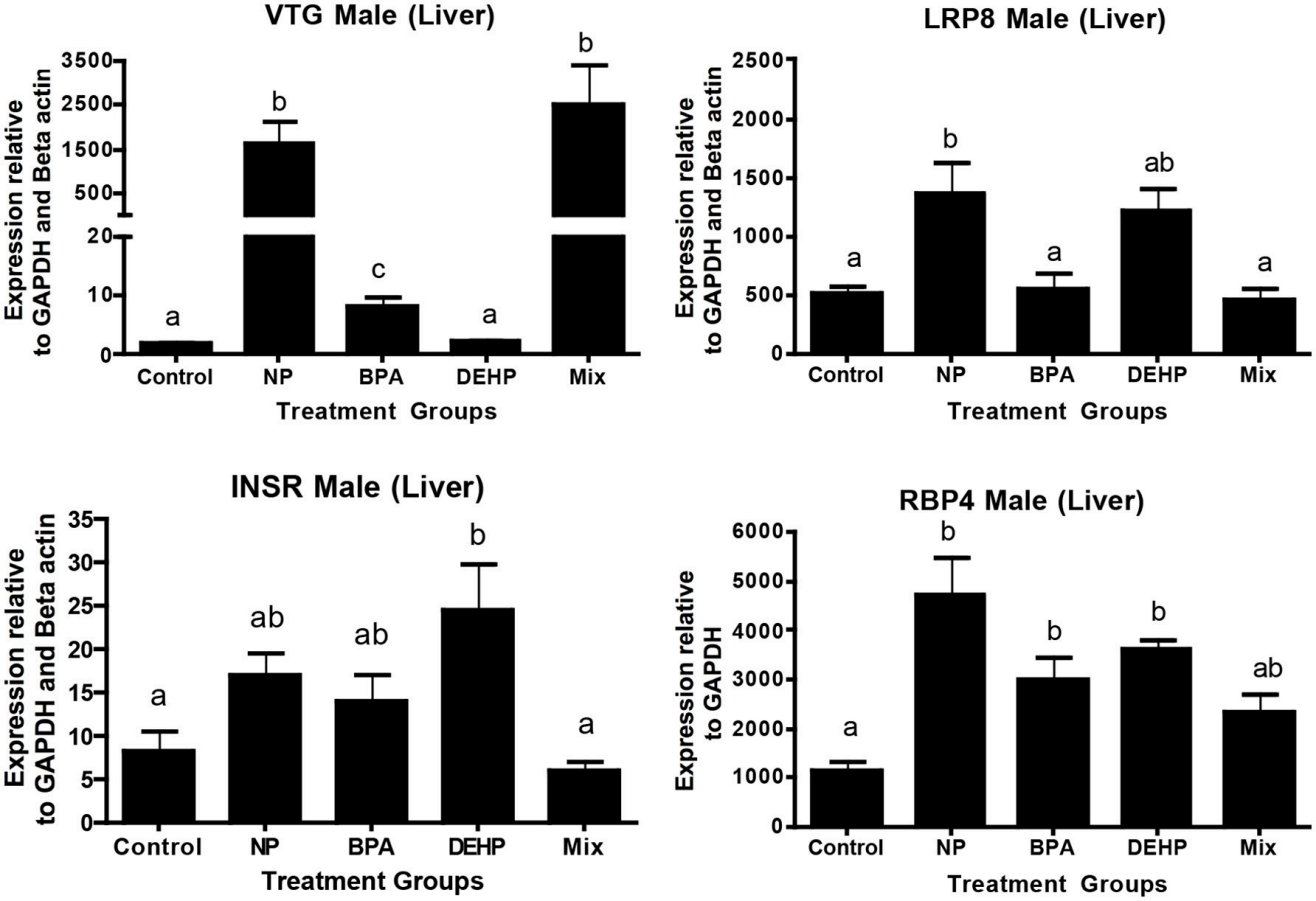
Validation of expression microarray results by quantitative real-time PCR (qPCR). Effects of 100 μg/L of NP, BPA, DEHP and the mixture of three chemicals on genes involved in lipid metabolism and transportation following 21 days exposure. The expression levels were measured using qPCR and normalized against the mean of GAPDH and β-actin (mean ± SEM). Different letters indicate a significant difference (ANOVA followed by Tukey Test, P < 0.05, n = 12).

**Figure 5:**
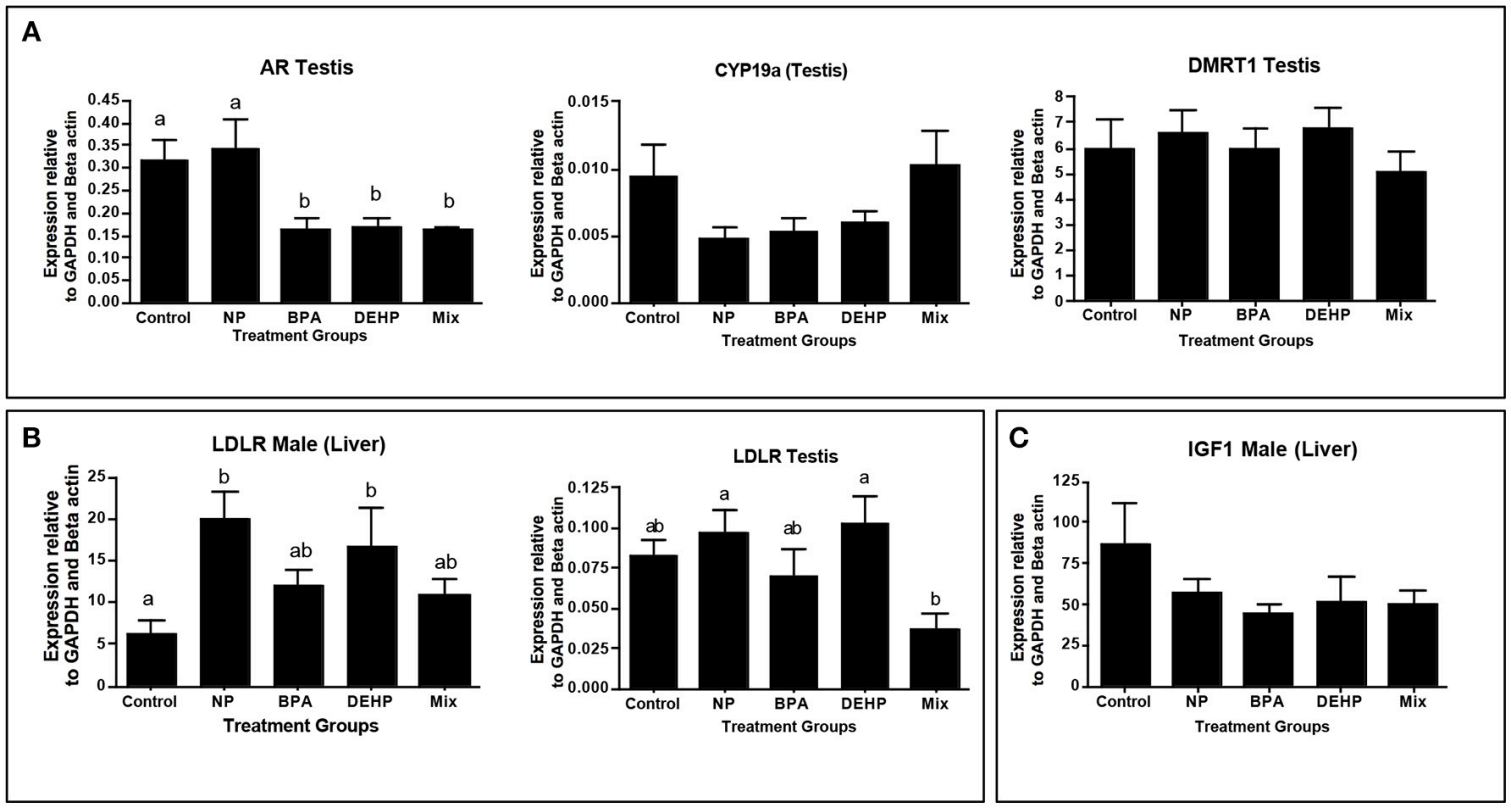
Confirmation of top regulators predicted by ingenuity pathway analysis (IPA) software, using quantitative real-time PCR (qPCR). Top regulators were predicted based on significantly regulated genes relative to control in microarray analysis. Genes were selected based of their function in the altered pathways: Androgen signaling **(A)**, PPAR/RXR Activation **(B)** and IGF1 signaling **(C)**. The expression levels were measured using qPCR and normalized against the mean of GAPDH and β-actin (mean ± SEM). Different letters indicate a significant difference (ANOVA followed by Tukey Test, P < 0.05, n = 12).

The expression microarray results demonstrated that Vtg1 is highly elevated by NP, BPA and the mixture compared to the untreated control (Table 5). These results were confirmed by qPCR (Figure 4). Microarray results also showed the same trend for Vtg3. DEHP did not have any significant effects on Vtg1 expression. The expression microarray data revealed that individual compounds and the mixture also disrupted a number of genes involved in lipid metabolism (Table S1). Some examples included various classes of Apolipoproteins and their binding proteins such as APOC1, APOA1, APOA4, LRP8, LRP1B, LDL-related protein, INSR, and HDLBP. LRP8 was significantly up-regulated by NP in the expression microarray data (Table 5) and qPCR (Figure 4). Our qPCR results also demonstrated that NP, BPA and DEHP significantly altered RBP4, but however, the expression microarray data showed that NP was the only treatment that up-regulated this gene. NP and DEHP significantly up-regulated LDLR in male liver but none of the treatment had a significant effect on LDLR in testis (Figure 5B). INSR showed down-regulation in response to DEHP and the mixture exposure from the expression microarray data (Table 3), but it was up-regulated by DEHP from qPCR analysis (Figure 4).

**Table 5:** Comparison of microarray analysis and qPCR results.

**Functional pathway**	**Gene name**	**qPCR results**				**Microarray analysis**			
		**NP**	**BPA**	**DEHP**	**Mixture**	**NP**	**BPA**	**DEHP**	**Mixture**
Immune response and detoxification	IFIT2	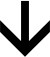	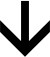	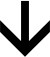	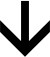	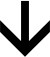	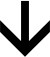	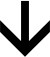	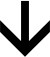
	RSAD2	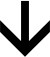	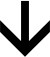	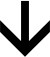	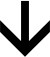	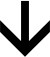	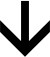	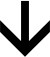	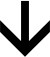
	IRF7	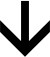	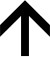	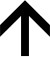	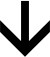	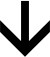	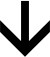	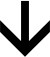	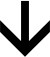
	GSTA1	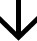	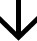	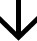	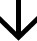	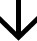	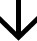	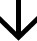	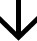
	CYP27B1	**-**	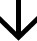	**-**	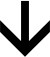	**-**	**-**	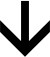	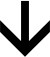
Lipid metabolism	Vtg1	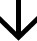	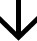	**-**	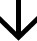	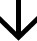	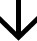	**-**	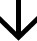
	LRP8	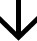	**-**	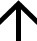	**-**	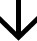	**-**	**-**	**-**
	RBP4	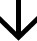	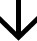	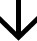	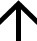	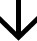	**-**	**-**	**-**
	INSR	**-**	**-**	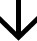	**-**	**-**	**-**	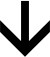	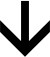

Relative changes in gene expression when compared to control group based on ANOVA followed by Tukey Test (P < 0.05, n = 12) for qPCR and P < 0.0001 and > 2 fold changes for microarray. Arrow direction indicates up or down regulation relative to control, bold and thin arrows represent significant and non-significant changes respectively and “-“ indicates no significant changes.

### Immune Response and Detoxification Pathway

**Figure 6:**
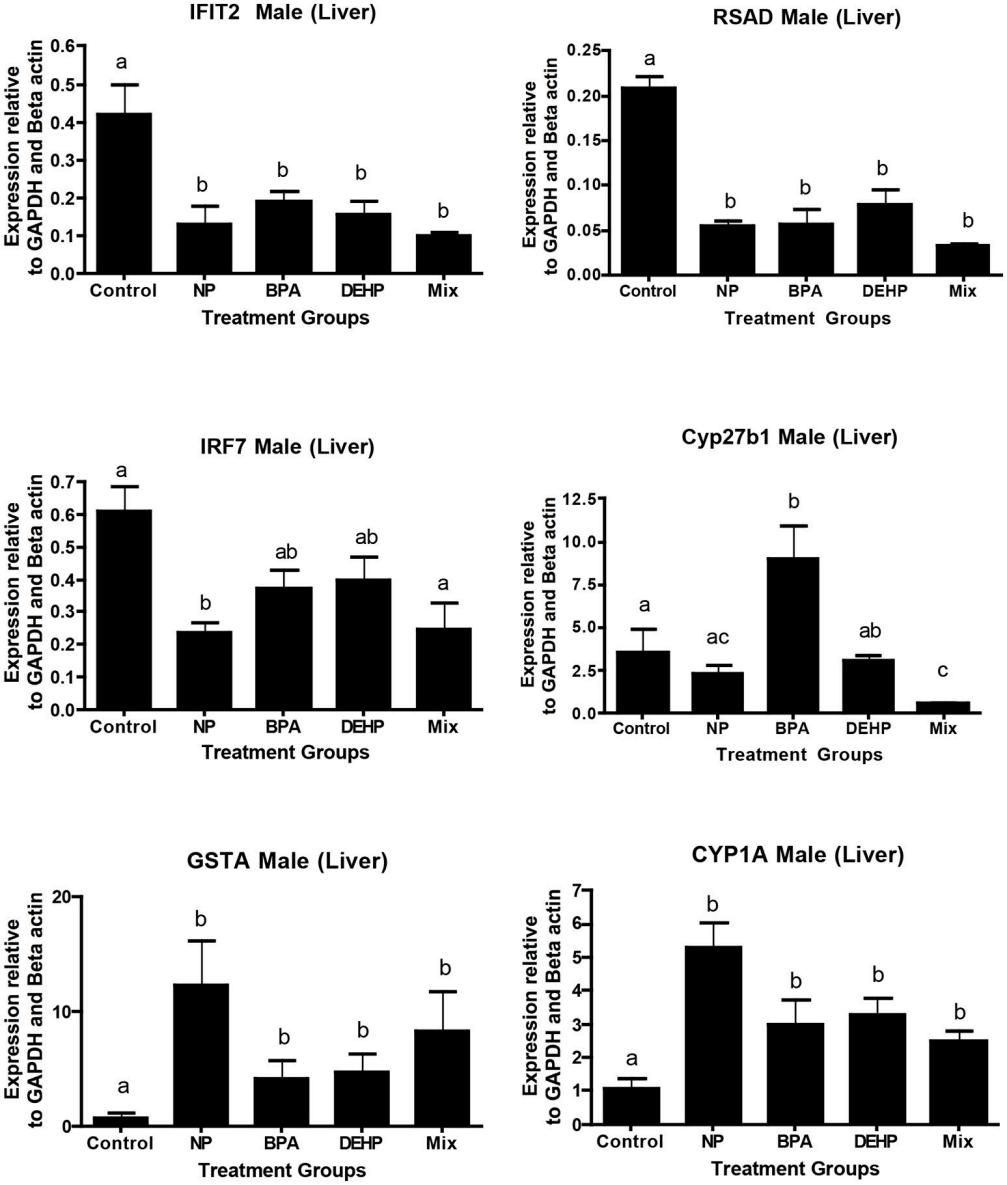
Validation of microarray results by quantitative real-time PCR (qPCR). Effects of 100 μg/L of NP, BPA, DEHP and the mixture of three chemicals on genes involved in the immune response and detoxification following 21 days exposure. The expression levels were measured using qPCR and normalized against the mean of GAPDH and β-actin (mean ± SEM). Different letters indicate a significant difference (ANOVA followed by Tukey Test, *P* < 0.05, *n* = 12).

A number of key elements in the immune response such as IFIT2, IRF7, and SRAD were highly inhibited by all of the four treatments from the expression microarray data and were confirmed by qPCR (Table 5). CYP27B, another gene with known roles in immune response, was down-regulated by the mixture treatment in both microarray and qPCR analysis. In contrast, GSTA1, which is involved in the detoxification pathway, was significantly elevated by all of the experimental treatments as demonstrated by expression microarray analysis and qPCR data (Figure 6)

### Represented Genes in Predicted Regulators and Canonical Pathways

IPA pathway analysis predicted the cascades of upstream transcriptional regulators that can explain the observed gene expression pattern. AhR and AR were among the top identified regulators. According to this analysis, the observed dysregulation in our dataset might be the result of the up-regulation of AhR and down-regulation of AR (Table 4). qPCR results consistent with the pathway analysis, demonstrated that CYP1A1, a marker for AhR activation, was significantly up-regulated by all four EDC exposures (Table 6) while AR expression was significantly down-regulated by BPA, DEHP and the mixture exposure (Figure 6). IGF1 signaling was one of the canonical pathways that were significantly affected by chemical exposure (Table 3). However, the expression level of IGF1, a key element in this pathway, was not influenced by any of the exposure groups (Figure 5). A possible explanation of this observation could be that other proteins and receptors in this pathway might be altered independently of IGF1 in response to EDC exposure as suggested in our expression microarray results.

**Table 6:** Comparison of top regulators predicted by IPA (fisher exact test p < 0.05) and gene expression of representative genes of the pathway using qPCR (ANOVA followed by Tukey Test, P < 0.05, n = 12).

**Predicted top regulators**	**Direction of pathway regulation (predicted by IPA)**				**Real-time PCR results**				
	**NP**	**BPA**	**DEHP**	**Mixture**	**Represented genes in the pathway**	**NP**	**BPA**	**DEHP**	**Mixture**
AhR	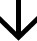	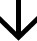	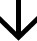	**-**	CYP1A1(liver)	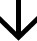	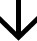	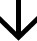	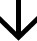
AR	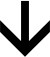	**-**	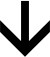	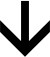	AR (testis)		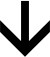	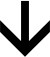	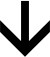
					DMRT1 (testis)	**-**	**-**	**-**	**-**
					Cyp19a1 (testis)	**-**	**-**	**-**	**-**

*Arrow direction indicates up or down regulation relative to control, bold and thin arrows represent significant and non-significant changes respectively and “-“ indicates no significant changes*.

## Discussion

In this study, we investigated the transcriptome changes associated with exposure to BPA, NP, DEHP and their tertiary mixture in the male FHM hepatic tissue. Using expression microarrays enabled us to identify the genes that were differentially regulated by each EDC treatment relative to the untreated control. We used expression microarray data to generate several hypotheses of the molecular mechanisms in response to EDC exposure rather than solely on the quantitative measure of changes in gene expression. In this context, observed variations in the transcriptome following exposure to these EDC contaminants can be used to identify potential candidate biomarkers for toxicity screening and determination of molecular pathways that are affected by these contaminants. The identified biomarkers could be used in toxicity assessments when a population is exposed to a variety of contaminants with a broad or perhaps unknown mechanism of action. More than 50% of the significantly altered genes were unique for each treatment. There is a potential to use some of these genes as a tool for biomonitoring specific contaminants and their potential health impacts.

A number of commonly regulated genes in response to all four EDC treatments such as IRF7, IFIT2, and RSAD were important elements in the inflammatory and immune response. Therefore, dysregulation of these genes in addition to their capacity for use as a molecular sensor of EDC exposure could reflect the impact of contaminants on health by adversely affecting the immune system. In this context, IRF members are central mediators in the regulation of innate immune responses (87). Expression of IRF members including IRF7 elevates in response to pathogens (88). IFIT proteins and RSAD2 are among interferon –stimulating genes that are induced by viral infection to suppress the virus infectivity (89, 90). Several studies showed that knocking down of these immune response genes could result in increased virus population in the body (89) or triggering breast cancer cell metastasis (91). Our findings in this study have demonstrated that IRF7, IFIT2, and RSAD are significantly down-regulated by all four EDC treatments. These results suggest that the EDC exposure may increase the fish susceptibility to pathogens and other stressors.

There is increasing evidence for the impacts of EDCs on the immune response (3, 92, 93). Our results are consistent with the reports that various class of environmental contaminants including BPA cause acute inflammatory response in zebrafish larvae by modulation of neutrophils and gene transcription, suggesting immunotoxicity (94). High concentrations of BPA were found to increase proliferation of a subset of immune cells including splenocytes, lymphocytes, thymocytes and B cells in goldfish or mice [Reviewed in (3)]. BPA could also exert negative impacts on the regulation of immune-associated genes in medaka (95) and juvenile common carp (*Cyprinus carpio*) (93). NP regulates various elements of the innate immune response in the Pacific oyster that was induced by bacterial infection (96). Exposure to 17alpha-ethylinestadiol (EE2) affected a large number of pathways associated with immune system in fathead minnow (97). In addition, AhR controls the expression of a number of genes involved in the immune response [Reviewed in (98)]. Therefore, the EDCs tested in this study may also act on the immune system via AhR.

Both innate and adaptive immune systems play a critical role in modulation of metabolic health. Exposure to environmental contaminants such as EDCs has been associated with a variety of physiological alterations, including reproductive impairment, metabolism dysregulation and immune dysfunction [Reviewed in (99)]. In our study, pathway analysis identified at least 10 signaling pathways that were altered by these treatments. These included pathways implicated in the Acute Phase Response, PI3K/AKT, EIF2 and IGF-1 signaling, PPAR/RXR activation, LXR/RXR activation, and NRF2-mediated oxidative stress response. Fathead minnows that were treated to municipal wastewater effluent (MWWE) for 21 days showed disruption in the pathways correlated with a variety of physiological alterations, including reproductive and immune dysfunction, cell adhesion, inflammation, estrogen receptor signaling and WNT signaling (100). In our study, GSTA1 was also altered by all four EDC treatments. It has been demonstrated that GSTs are upregulated in many fish species exposed to oil and hydrocarbons (101, 102).

The PPAR family of nuclear receptors has been associated with a number of inflammatory disease states. PPARγ agonists have been shown to suppress the expression of proinflammatory cytokines in the spinal cord and BPA may alter the regulatory/anti-inflammatory axis through interaction with PPARs [Reviewed in (3)]. Exposure to weakly estrogenic effluent reduced immune system activity such as reductions in the number of circulating lymphocytes, but increased nonspecific immune activation in fathead minnow (103).

Lipid molecules through interactions with a large family of nuclear receptors regulate signaling cascades involved in development, cellular differentiation, and metabolism. PPARs exert the majority of their effects as heterodimers with the retinoid x receptor (RXR) and are some of the key regulators of lipid and carbohydrate metabolism that also regulate inflammatory responses [Reviewed in (104)]. In African sharptooth catfish (*Clarias gariepinus*), exposure to DEHP led to dysregulation of PPAR gene expression and protein levels which consequently affects lipid homeostasis (105). Expression of genes related to lipid accumulation, oxidative stress and proteolysis were altered in female rare minnow (*Gobiocypris rarus*) following exposed to different doses of BPA in a non-monotonic pattern (38).

Exposure to BPA in zebrafish resulted in dysregulation of fatty acid synthesis pathway, leading to the production of cholesterol esters and conversion to lipid droplets (106). Dietary administration of NP, *t-*OP or BPA individually and in a mixture altered the expression of genes involved in lipid metabolism resulting in hepatic steatosis in juvenile gilt-head sea bream (*Sparus aurata*) (107, 108). In adult male gilt-head sea bream dietary exposure to BPA elevated the amounts of lipids and triglycerides in the liver and dysregulated the levels of endocannabinoids (EC) (109). Male burbot fish (Lota lota) from the lakes contaminated with POP (persistent organic pollutant) showed that FXR/RXR and LXR/RXR activation, and the NRF2-mediated oxidative stress response were the most significantly affected pathways. The authors also discovered that differentially-regulated genes were significantly involved in lipid and vitamin A metabolism (110). PPAR, a nuclear receptor involved in the regulation of lipid homeostasis, was also identified as upstream key elements involved in the response to POP. Thus, exposure to EDC contaminants can potentially exert adverse health impact by producing harmful compounds in the body through activation of inflammation, (oxidative) stress, lipid peroxidation, and other natural processes (111).

The presence of complex chemical mixtures in the environment and our limited knowledge on mechanisms of toxicity and the interaction of chemicals in a mixture are the main challenges for design methodology guidelines to evaluate the potential ecological and human health risk assessment (64, 112). Several mathematical based models have been designed to predict the toxicity effects of EDC mixtures [Reviewed in (52, 113)]. However, the toxicokinetic interactions of mixture components, and their physiological and metabolism pathways that are altered by each compound in a mixture and also their adverse outcome at the level of the biological organization often have been neglected in these studies (113). In the present study, the gene expression pattern of the mixture-exposed FHM was found to be different from individually exposed chemicals. Exposure to a mixture of BPA, DEHP and NP led to an integrated stress response by inhibiting the Acute Phase Response Signaling and activating the EIF2 Signaling, which are common adaptive mechanisms (114). This stress response was detected in a greater extent in the EDC mixture compared to the individual exposures. While we acknowledge that the mixture in this study is not completely representative of the environmental mixture, the information obtained in this study is important for the overall understanding of toxicokinetic interactions of contaminants when they are mixed.

With an increase in emerging chemicals to the environment, there is a growing demand for ecological risk assessors to evaluate each chemical and in combination more accurately in a shorter time while using less resources and animals (115). Advances in biological science and the applications of Omics technologies along with advances in computational analytical tools may provide valuable information on mechanistic pathways that link molecular events to adverse outcomes at the ecological level. Transcriptomics combined with computational pathway analysis could provide some useful information on the key events at the cellular or organ level which facilitates predictive outcome effects in organisms or population (59, 68, 115–117). The Omics approach could characterize the biochemical pathways targeted by the contaminants and the common regulators perturbed by the chemical exposure which can be subsequently applied to ecotoxicology research (66).

Some molecular initiating events caused by particular classes of chemicals and their adverse outcomes are well-documented. For instant, the chemicals that activate the xenobiotic-metabolizing enzymes through binding to AhR, can result in reproductive impairment, cancer, perturbation in immune response, and early-life-stage mortality [Reviewed in (118)]. Estrogen receptor (ER) agonists are another example where binding of an estrogen-mimicking chemical to the ER resulted in changes in expression of estrogen-responsive genes, alterations in plasma sex steroid and vitellogenin concentrations at the cellular level, and gonadal abnormalities (such as intersex) at the organ level. These key events lead to changes in secondary sex characteristics and reproductive behavior and consequently may influence exposed populations (115). However, further study on time and dose dependent effects of EDC's is required to fill the gap between traditional toxicity biomarkers (reproduction and growth) and transcriptome data. It is necessary to obtain basic knowledge about biological systems and the molecular mechanisms by which chemicals disturb them, to improve the ability to predict the potential adverse effects of compounds and to identify the compounds that caused observed effects in the environment (115, 119). Network analysis makes it possible to identify the adverse outcome pathways (AOPs) and to predict the mechanisms underlying chemical toxicity and its link to human diseases and endocrine disorders (116).

Useful molecular biomarkers to evaluate biological responses to environmental contaminants need to be robust, reliable, and repeatable in various laboratories. Using transcriptome datasets generated from six independent laboratories, which exposed male fathead minnows to 17alpha-ethylinestadiol (EE2), it was demonstrated that EE2 regulates processes related to lipid metabolism and peroxidation, innate immune response, and IGF signaling in the liver of male fathead minnow (120). Results of our study also revealed these pathways were altered in response to chemical exposure. However, the 18 commonly regulated transcripts shown in our study are not among the identified estrogen-responsive transcripts that were found in the EE2 exposed fish. This is not surprising considering the chemical property of our experimental compounds. BPA and NP are traditionally considered as estrogen mimicking compounds and DEHP is known as an androgen receptor antagonist. It should be noted that these compounds are not only estrogenic and are known to interact with different types of hormone receptors and consequently alter distinct signaling pathways, when compared to EE2. In this regard, overlap of specificity for BPA and DEHP were shown in a number of previous studies (23, 49).

Using GSEA (gene set enrichment analysis)-based tools makes it possible to use non-human model organisms to make a potential connection between environmental conditions that lead to differentially-expressed gene sets and human disorders. This approach evokes hypotheses regarding circumstances that lead to potential onset of human diseases and dysregulation of biological processes. Also, comparing the gene expression profile of the model organism after exposure to a contaminant to a human gene set involved in a particular pathway and function, can make it possible to predict the adverse impacts of a given treatment to humans (69).

In summary, our results demonstrate that exposure to BPA, DEHP, NP and a mixture of all these EDCs can trigger distinct gene expression patterns in fathead minnow. Despite the differences, all these treatments regulate many key elements in the immune response pathway that might result in increasing susceptibility of fish to pathogens and other stressors. Novel canonical pathways and causal networks were also identified in response to each of the contaminants as well as the mixture. Our results provide information about the molecular mechanisms of the chemicals in a mixture compared to the individual exposure. The EDC mixture inhibited Acute Phase Response Signaling and activated the EIF2 Signaling, which are common adaptive pathways functioning in the integrated stress response (114). This stress response was not observed in the individual treatments. We also investigated a number of relevant genes affected by all the EDC treatments, and the expression of most of these genes detected by expression microarrays were validated by qPCR. We propose that these genes could potentially act as biomarker candidates in screening for the presence and biological effects of these contaminants.

Using gene set enrichment analysis of transcriptome data allowed the use of a non-human model organisms to make a potential connection between environmental conditions that lead to differentially-expressed gene expression and human disorders. Network analysis made it possible to predict the potential mechanisms underlying chemical toxicity and its potential link to human diseases and disorders caused by contaminants.

## Author Contributions

AZ and HH designed research. AZ performed research. AZ, DH, and PG analyzed data. GC provided intellectual input on microarray analysis. AZ and HH wrote the paper. HH provided funding, oversight, intellectual input on experimental design and data analysis.

### Conflict of Interest Statement

The authors declare that the research was conducted in the absence of any commercial or financial relationships that could be construed as a potential conflict of interest. The handling Editor declared past co-authorships with one of the authors HH.
