# Supplementary material for: Differential Hepatic Gene Expression Profile of Male Fathead Minnows Exposed to Daily Varying Dose of Environmental Contaminants Individually and in Mixture
Source: Front Endocrinol (Lausanne). 2018 Dec 10;9:749. doi: 10.3389/fendo.2018.00749 (PMC6295643; doi:10.3389/fendo.2018.00749)
Supplement: Supplementary file 1 [file Table_1.DOCX]

**Supplementary Data**

Table S1. Gene annotations and GO terms for the transcripts significantly regulated by all three individual compounds. Gene annotations and GO terms were provided by EcoArray.

| Gene description | **Gene ID** | **NCBI Accession** | **GO terms** |
| --- | --- | --- | --- |
| Mucin 1, Transmembrane | C14orf173 | AF221160 | - |
| Angiopoietin-like 3 | ANGPTL3 | AF379604 | GO:0008372; cellular component unknown  GO:0005554; molecular function unknown |
| Keratin 4 / Type II Cytokeratin / Keratin 8 | KRT8 | BC063955 | GO:0005882; intermediate filament  GO:0005198; structural molecule activity |
| Glutathione S-transferase A1 | GSTA1 | BC060914 | GO:0016740; transferase activity |
| Lactase | LCT | Z27167 | GO:0005975; carbohydrate metabolism  GO:0005624; membrane fraction activity  GO:0015926; glucosidase activity |
| Interferon Regulatory Factor 7 | IRF7 | AY177629 | GO:0006350; transcription |
| GO:0003677; DNA binding |  |  | - |
| Radical S-adenosyl Methionine Domain Containing 2 | RSAD2 | AY303809 | GO:0005506; iron ion binding |
| GO:0003824; catalytic activity |  |  | - |
| Chromosome 10 Open Reading Frame 56 | C10orf56 | XM_356713 | GO:0003676; nucleic acid binding |
| Similar To Interferon-inducible Protein IFI56 | IFIT2 | AY267212 | - |
| Chemokine Ligand 20 | CCL19 | NG_003082 | GO:0006935; chemotaxis  GO:0009615; response to virus  GO:0006874; calcium ion homeostasis  GO:0006954; inflammatory response |
| Similar To Prostate Stem Cell Antigen Precursor | PLAUR | AP004509 | GO:0007596; blood coagulation  GO:0007166; cell surface receptor linked signal transduction GO:0006935; chemotaxis  GO:0005515; protein binding |
| Spastic Ataxia Of Charlevoix-Saguenay | SACS | BX005194 | GO:0006457; protein folding  GO:0050750; low-density lipoprotein receptor binding GO:0031072; heat shock protein binding |
| Si:xx-51f19.2 | STAT1 | AY242386 | GO:0006355; regulation of transcription, DNA  GO:0005509; calcium ion binding |
| Zgc:65956 | PHGDH | BC056334 | GO:0008152; metabolism  GO:0006564; L-serine biosynthesis  GO:0004617; phosphoglycerate dehydrogenase activity |
| DnaJ Homolog, Subfamily A, Member 4 | DNAJA4 | XM_345525 | GO:0006457; protein folding  GO:0046872; metal ion binding  GO:0008270; zinc ion binding  GO:0031072; heat shock protein binding |
| Similar To Unknow Protein | - | AC144429 | - |
| Synaptonemal Complex Protein 1 | SYCP1 | BX005150 | GO:0051301; cell division  GO:0007283; spermatogenesis  GO:0007126; meiosis  GO:0007131; meiotic recombination  GO:0007049; cell cycle |
| Im:7141452 | 0 | AL592144 | - |
| Hypothetical Protein FLJ40311 | HSF5 | AL596086 | GO:0006350; transcription  GO:0006986; response to unfolded protein  GO:0006457; protein folding  GO:0006355; regulation of transcription, DNA-dependent |
| dopamine receptor [Takifugu rubripes] | 0 | AL672032 | - |
| EF-hand Domain Containing 2 | EFHC2 | BX572627 | GO:0005509; calcium ion binding |
| Heat Shock Protein 83 | HSP90AA1 | U75687 | GO:0007015; actin filament organization  GO:0007283; spermatogenesis  GO:0008595; determination of anterior/posterior axis, embryo  GO:0006457; protein folding  GO:0045187; regulation of circadian sleep/wake cycle, sleep |
| ATP Binding / ATP-dependent Helicase/ Helicase/ Nucleic Acid Binding | DDX43 | BX890626 | GO:0005739; mitochondrion  GO:0004386; helicase activity  GO:0003676; nucleic acid binding |
| Myelin Basic Protein | MBP | CR693650 | - |
| Daz-like Gene | DAZL | BC076423 | GO:0006445; regulation of translation  GO:0006417; regulation of protein biosynthesis  GO:0030154; cell differentiation  GO:0007283; spermatogenesis  GO:0048477; oogenesis |
| Complement Factor H | CFH | BX005398 | GO:0000004; biological process unknown |
| Similar To FLJ44216 Protein | C5orf25 | AL935334 | - |
| Stromal Antigen 2 | STAG3 | AJ007798 | GO:0051301; cell division  GO:0007126; meiosis  GO:0007067; mitosis GO:0007049; cell cycle |
| Chromosome 16 Open Reading Frame 50 | CCDC135 | BC030661 | - |
| Zebrafish DNA sequence from clone CH211-276E22 in linkage group 4, | 0 | BX855588 | - |
| Vasa Homolog | DDX4 | AF479820 | GO:0008406; gonad development  GO:0004386; helicase activity  GO:0003676; nucleic acid binding |
| Sb:cb26 | C3 | AY374472 | GO:0006956; complement activation  GO:0006955; immune response  GO:0001798; positive regulation of type IIa hypersensitivity GO:0045087; innate immune response  GO:0006954; inflammatory response |
| Cytochrome B | 0 | AF352263 |  |
| Serine Peptidase Inhibitor, Clade I, Member 2 | SERPINI1 | AL845366 | GO:0007422; peripheral nervous system development GO:0007417; central nervous system development |
| hypothetical protein Francci3DRAFT_1939 [Frankia sp. CcI3] | 0 | AC118750 | - |
| Fetuin Beta | FETUB | CR735002 | GO:0005576; extracellular region  GO:0004869; cysteine protease inhibitor activity |
| Ornithine Decarboxylase 1 | ODC1 | BC063932 | GO:0006596; polyamine biosynthesis  GO:0003824; catalytic activity |
| Amyloid Beta Precursor Protein | APP | AJ315639 | GO:0016021; integral to membrane  GO:0005488; binding  GO:0004867; serine-type endopeptidase inhibitor activity |
| Choline Kinase Alpha | CHKA | BX548052 | GO:0006869; lipid transport  GO:0006629; lipid metabolism  GO:0016301; kinase activity |
| Calcium Binding Protein 39-like | CAB39L | BX294379 | GO:0005509; calcium ion binding  GO:0005488; binding |
| Flavin Containing Monooxygenase 5 | FMO5 | AY394974 | GO:0006118; electron transport  GO:0005783; endoplasmic reticulum  GO:0016021; integral to membrane  GO:0031227; intrinsic to endoplasmic reticulum membrane |
| Apolipoprotein A-IV | APOA4 | BC076032 | GO:0006869; lipid transport  GO:0042157; lipoprotein metabolism  GO:0005576; extracellular region  GO:0008289; lipid binding |

**Table S2.** Regulatory networks activated in the liver of male fathead minnow in response to exposure to NP. The Regulatory network was generated using Ingenuity Pathways Analysis (IPA) software.

| **Regulators** | **Target Molecules in Dataset** | **Score** | **Focus Molecules** | **Diseases & functions** |
| --- | --- | --- | --- | --- |
| GPAM,JAK1,SIN3A,TSC2 | BCL6,BIRC5,CDKN1A,CYP27B1,ESR1,G6PC,GADD45B,GSTP1,HSPA5,MCL1,MMP9,PDGFRB,PLIN2,SCD,SOCS3,STAT1 | 50 | 34 | Arthropathy,  Concentration of fatty acid,  Hepatic steatosis,  Nephritis,  Rheumatic disease,  Steroid metabolism |
| MMP9,Nr1h,PTH | ACSL3,APOC1,APOE,C3,CDKN1A,CYP7A1,FASN,G6PC,GADD45A,GCK,IL6R,JUN,LPL,PLTP,SOCS3 | 46 | 31 | Efflux of phospholipid,ingestion by mice,Nephritis,transport of lipid |
| Esrra,FOXA2,HNF1A | ACSL5,AHSG,APOA4,APOB,C3,CYP7A1,FABP1,FABP2,G6PC,GATM,GC,GCK,INSR,KNG1,LPL,NR1H4,PFKFB2,SCD,SOCS3,SST | 35 | 26 | Transport of lipid,uptake of monosaccharide |
| AhR,PIAS1 | ABCB11,ACOX1,APP,CDKN1A,ESR1,FASN,FOS,JUNB,SCD | 32 | 24 | Accumulation of lipid,  Weight gain |
| IL3 | CDKN1A,CXCR4,HBEGF,HSP90B1,HSPA5,JUN,MCL1,MDM2,PIM1,PIM2,SOCS1,SOCS3 | 31 | 25 | Cell viability of tumor cell lines |
| PPARG | ACSL5,APOE,FABP1,FABP2,LPL,PLIN2,RBP4,SCP2 | 30 | 24 | Transport of lipid |
| PPARG | ACOX1,APOE,FABP2,G6PC,ODC1,SAT1,SOCS3 | 30 | 23 | Mass of liver |
| NFATC2 | CDKN1A,ENPP2,MDM2,SOCS1,SOCS3,STAT1 | 29 | 23 | Rheumatic disease |
| Nr1h | APOC1,APOE,CYP7A1,LPL,PLTP,SCD | 28 | 25 | Flux of lipid |
| PARP1 | FASN,FKBP5,FOS,MDM2,POLA1,SOCS1,STAT1 | 28 | 22 | Rheumatic disease |
| PIAS1 | CDKN1A,GADD45A,MCL1,MDM2,STAT1 | 26 | 21 | Quantity of leukocytes |
| Nr1h | APOE,C3,GCK,SCD | 24 | 20 | Feeding |
| SAMSN1 | HBEGF,SOCS1,SOCS3 | 24 | 20 | Uptake of monosaccharide |
| KRAS | ALDOA,B2M,BIRC5,CDKN1A,CLU,FBN1,FOS,HSPA5,MDM2,STAT1 | 24 | 20 | Rheumatic disease |
| LCK | ANXA1,CD69,FOS,MMP9 | 22 | 19 | Arthritis |
| CSF2 | BIRC5,CDKN1A,CXCR4,HBEGF,ID2,MCL1,MDM2,PIM1,PIM2,SGK1,SOCS1,SOCS3,TNFRSF1A | 22 | 19 | Cell viability of tumor cell lines |
| ERBB2 | CDKN1A,CLU,ESR1,HBEGF,JUN,MDM2,MMP9 | 19 | 17 | Nephritis |
| IL5 | CDKN1A,GADD45A,HBEGF,MMP9,PIM1,SOCS1 | 19 | 17 | Nephritis |
| BDNF | APP,CDKN1A,CXCR4,EGR1,FOS,MBP,PLG,SST | 19 | 17 | Quantity of leukocytes |
| IRF1 | CDKN1A,MMP9,SOCS1 | 18 | 17 | Nephritis |

**Table S3**. Regulatory networks activated in the liver of male fathead minnow in response to exposure to BPA. The Regulatory network was generated using Ingenuity Pathways Analysis (IPA) software.

| **Regulators** | **Target Molecules in Dataset** | **Score** | **Focus Molecules** | **Diseases & Functions** |
| --- | --- | --- | --- | --- |
| FOXC2,MAP4K4,MEDAG,MLXIPL,NR1I3,PPARGC1A,RXRA | ACAA2,ACADM,ACLY,ACOX1,ACSL3,ACSL5,ANGPTL3,APP,ATP5J2,C3,CXCR4,FABP1,FABP2,FASN,G6PC,GCK,H6PD,INSR,KNG1,LIPA,LPL,NR1H4,OGDH,PDGFRB,PNPLA2,POR,SAT1,SOCS3 | 53 | 35 | Binding of cells,  Mass of liver,  Metabolism of nucleotide,  Oxidation of fatty acid |
| BCL6,CNTF,EIF2AK2,IFNA2,IFNAR1,INSIG1,LIF,PPARG,PRKCD,SAMSN1,STAT,STAT1 | ACLY,ACOX1,ACSS2,APP,ATF3,B2M,C3,CCR7,DSPP,FASN,FOS,IL10RB,IL7R,INSR,JUN,JUNB,LIPA,LPL,MGLL,PPARGC1A,PTTG1,SCP2,SOCS1,SOCS3,SST,TFCP2L1,TNFRSF1A,XBP1 | 39 | 27 | Conversion of fatty acid,  Morphology of digestive system |
| ACKR2 | IFIT2,IRF7,RSAD2,STAT1 | 38 | 28 | Viral infection |
| PARP1 | FASN,JUN,MDM2,POLA1,SOCS1,SOCS2,STAT1 | 34 | 25 | Viral infection |
| SP1 | ANKH,ATF3,CBS/LOC102724560,ESR1,FOS,INSR,JUN,LIPA,LPL,PTTG1,SOCS1,STAT1 | 32 | 24 | Morphology of digestive system |
| DOCK8 | IFIT2,IRF7,RSAD2,SOCS1,STAT1 | 32 | 24 | Viral infection |
| Pro-inflammatory Cytokine | HSPA5,LIPG,NR1H4,SLC6A4 | 30 | 23 | Binding of cells |
| mir-15 | APP,CD69,SLC6A4 | 30 | 23 | Binding of cells |
| IFNA2 | ANXA1,B2M,C1S,CXCL11,IFI35,MDM2,RSAD2,STAT1,XBP1 | 26 | 21 | Infection by RNA virus |
| Ifn | B2M,CD69,CXCL11,IRF7,RSAD2,SOCS1,STAT1,TFRC | 25 | 20 | Viral infection |
| DDX58 | IFI35,IFIT2,IRF7,RSAD2,SOCS1,SOCS3,STAT1 | 24 | 20 | Viral infection |
| EPO | CCL19,CLGN,CXCR4,JUN,PDLIM2,TFRC | 23 | 20 | Binding of cells |
| Ifnar | B2M,C3,IFI35,RSAD2,STAT1 | 23 | 19 | Infection by RNA virus |
| Ifnar | C3,IFI35,RSAD2,STAT1 | 23 | 19 | Infection of cells |
| IRF1 | B2M,CASP2,IFI35,IFIT2,IRF7,RSAD2,SOCS1,STAT1 | 22 | 19 | Viral infection |
| Interferon alpha | C3,CCL19,CD209,CD69,CXCL11,INSR,SLC6A4 | 21 | 18 | Binding of cells |
| TRIM24 | IFI35,IFIT2,IRF7,PARP12,SOCS1,SOCS2,STAT1,TRAFD1 | 21 | 18 | Viral infection |

**Table S4**. Regulatory networks activated in the liver of male fathead minnow in response to exposure to DEHP. The Regulatory network was generated using Ingenuity Pathways Analysis (IPA) software.

| **Regulators** | **Target Molecules in Dataset** |  |  | **Diseases & Functions** |
| --- | --- | --- | --- | --- |
| CEBPA,DDX58,IFNA2,IFNAR1,IL27,IRF7,MAVS,NFATC2,PIAS1,SAMSN1 | ABCB11,APOB,C3,EGR1,GSTP1,IL10RB,IL6R,INSR,MMP9,NR1H4,SCD,SOCS1,SOCS3,STAT1,TNFRSF1A | 51 | 34 | Fibrosis of liver,  Focal necrosis of liver,  Inflammation of liver |
| DDX58,FBXO32,IL3,IL6ST,JINK1/2,MAP4K4,MAVS,MGEA5,NFATC2,SAMSN1 | ACSL5,CASP2,CDKN1A,CXCR4,CYP7A1,DNAJC3,EIF2AK3,ENPP2,FASN,FLNA,FOS,GADD45A,HBEGF,HPX,HSP90B1,HSPA5,IL6R,IRF7,JUN,MCL1,MDM2,ODC1,PDHA1,PHGDH,PIM1,PIM2,PLG,PPAP2B,SCD,SLC3A2,SOCS1,SOCS3,STAT1,TNFRSF1A,XBP1 | 44 | 29 | Cell viability of tumor cell lines,  Fibrosis of liver,  Metabolism of triacylglycerol,  Organismal death,  Proliferation of breast cancer cell lines |
| EHHADH,HSD17B4,NR0B2,PPARÎ±-RXRÎ± | ABCB11,ACAA1,ACADM,ACOX1,APOA1,CYP7A1,EGR1,FASN,LPL,NR1H4,PPARGC1A,SCD,SCP2 | 42 | 29 | Inflammation of liver,  Oxidation of lipid,  Quantity of insulin in blood,  Secretion of cholesterol |
| CEBPA,JINK1/2,MAP4K4,MAPK9 | ACSL5,APOB,BHMT,C3,CYP7A1,DGAT2,FASN,LPL,PLIN2,SCD | 39 | 27 | Storage of lipid,  Synthesis of triacylglycerol |
| PIAS1,TSC2 | ABCB11,BIRC5,CDKN1A,HSPA5,MCL1,MDM2,SCD,STAT1 | 33 | 24 | Cellular homeostasis,  Necrosis of liver,  Secretion of molecule |
| JINK1/2,MAP4K4 | ACSL5,CYP7A1,FASN,PLG,SCD,TNFRSF1A | 32 | 25 | Differentiation of cells,  Metabolism of triacylglycerol,  Organismal death |
| IL3,IL6ST | CDKN1A,CXCR4,FASN,HBEGF,HSP90B1,HSPA5,IL6R,JUN,MCL1,MDM2,PIM1,PIM2,SOCS1,SOCS3,TNFRSF1A | 29 | 22 | Cell viability of tumor cell lines,  Replication of rna virus |
| ATF4,TSC2,Vegf | BIRC5,EGR1,FASN,HBEGF,HSPA5,JUN,MCL1,PLAUR,SOCS3 | 29 | 22 | Apoptosis of prostate cancer cell lines |
| GHR,JAK1,SLC13A1 | ACSL5,CDKN1A,FABP2,HSPA5,IGFBP1,IGFBP2,SCD,SOCS3,SST,STAT1,XBP1 | 26 | 21 | Proliferation of tumor cell lines |
| Ifnar,PRDM1 | B2M,BCL6,C3,CFH,FGG,FOS,IFIT2,MCL1,MMP9,XBP1 | 26 | 21 | Apoptosis of liver,  Cell death of liver cells,  Movement disorders |
| GHR,Hmgn3,MAP4K4,miR-217-5p (and other miRNAs w/seed ACUGCAU) | ACAA2,ACOX1,ACSL5,AHSG,APOA1,CDKN1A,DLAT,FASN,FGA,IGFBP1,IGFBP2,KNG1,LPL,OGDH,PPARGC1A,SCD,SCP2,SERPINA1,SOCS3,SST | 26 | 21 | Cell death of tumor cell lines,  Cell movement of tumor cell lines,  Fatty acid metabolism,  Proliferation of tumor cell lines,  Secretion of molecule,  Synthesis of triacylglycerol |
| CDC42,miR-1-3p (and other miRNAs w/seed GGAAUGU) | AP3D1,ARID1A,ATF3,ESR1,FOS,JUN,PIM1,PNP,PPIB | 24 | 20 | Apoptosis of blood cells,  Cellular homeostasis |
| CDKN1A,MED1 | APOB,APP,MDM2,MMP9 | 23 | 19 | Mortality |
| EGF,HNF4A | APOE,CBS/LOC102724560,GSTP1,HPX,PDGFRB,PLAUR,PLG,SCD,SOCS3,STAT1 | 23 | 19 | Fibrosis of liver |
| CEBPB,CSF2,HGF,LIF,NFE2L2,NFKBIA | ABCB11,APOA1,APOB,ATF3,AZGP1,BHMT,C3,CASP2,CCR7,CDKN1A,CLU,CXCR4,CYP27B1,DNAJC3,EGR1,FLNA,FOS,GHR,HBEGF,HSP90B1,ID2,IL7R,INSR,JUN,JUNB,KRT8,LPL,MCL1,MDM2,MEF2C,MMP9,NEUROD1,PLA2G15,PLG,PPARGC1A,PPP1R15A,PTTG1,SOCS1,SOCS3,SST,SYCP1,TNFRSF1A,XBP1 | 22 | 19 | Morphology of body cavity |
| PTEN | ACOX1,CDKN1A,ESR1,FOS,IGFBP2,MDM2,PDGFRB | 21 | 18 | Transformation of fibroblast cell lines |
| Cg,Growth hormone,Ifnar | B2M,C3,CLU,EGR1,FOS,G6PC,IFIT2,IGFALS,JUN,LPL,MMP9,PLAUR,SACS,SGK1 | 19 | 19 | Movement disorders |
| PTGER2 | APP,CCR7,CXCR4,EGR1,MMP9,PIM1 | 19 | 19 | Quantity of blood cells |
| IL6 | ABCB11,ACOX1,APOA1,APOB,APOE,ATF3,BCL6,C3,CCR7,CDKN1A,CLU,CXCR4,EGR1,FGA,FLI1,FOS,ID2,IL7R,JUN,JUNB,KRT8,LIG1,LPL,MCL1,MMP9,NR1H4,PLG,PPP1R15A,PTTG1,SOCS1,SOCS3,SST,TNFRSF1A,XBP1 | 18 | 16 | Morphology of body cavity |
| XBP1 | APOA1,APP,EIF2AK3,ESR1,HERPUD1,HSP90B1,HSPA5,PPIB,SERPINA1 | 18 | 16 | Cellular homeostasis |
| Growth hormone | EGR1,FASN,GHR,LPL,MMP9,PPP1R15A,SGK1,SOCS1,SOCS2,SOCS3 | 18 | 16 | Viral infection |
| NR0B2 | ABCB11,APOA1,CYP7A1,EGR1,G6PC,NR1H4,PPARGC1A,SCD | 18 | 16 | Transport of molecule |
| AR | AQP3,C3,CDKN1A,EGR1,FKBP5,GSTP1,HSPA5,ODC1,PFKFB2,SGK1,STAT1 | 18 | 16 | Cell survival |
| MED1 | APOB,BIRC5,CDKN1A,GADD45A,GHR,GSTP1,MDM2 | 18 | 18 | Quantity of leukocytes |
| CEBPA | APOA4,C3,CYP7A1,INSR,LPL,NR1H4,PLIN2,PPARGC1A | 17 | 17 | Uptake of lipid |
| CEBPB | C3,CYP27B1,INSR,PLG,SST,TNFRSF1A | 51 | 34 | Release of lipid |
| NFATC2 | CDKN1A,CISH,MDM2,SOCS1,SOCS3,STAT1 | 44 | 29 | Quantity of mononuclear leukocytes |
| PIAS1 | CDKN1A,GADD45A,MCL1,MDM2,STAT1 | 42 | 29 | Quantity of cells |
| Irgm1 | BUB1,ID2,KIF20A,UBE2C | 39 | 27 | Proliferation of tumor cell lines |
| LTBR | CCL19,CLU,ENPP2,SERPINA1 | 33 | 24 | Cell movement of tumor cell lines |
| IRF1 | B2M,CASP2,CDKN1A,IRF7,MMP9,SOCS1 | 32 | 25 | Morphology of body cavity |
| CLDN7 | APOE,C3,SLCO2A1 | 29 | 22 | Uptake of lipid |
| let-7 | BUB1,ID2,NR1H4 | 29 | 22 | Proliferation of breast cancer cell lines |
| IRF3 | B2M,CCL19,CD69,IRF7,JUNB,MMP9,PNP,RSAD2,STAT1 | 26 | 21 | Differentiation of cells |
| IFNA2 | ANXA1,B2M,C1S,CXCL11,IFI35,MDM2,MMP9,RSAD2,STAT1,XBP1 | 26 | 21 | Infection by RNA virus |
| SASH1, DOCK8 | IRF7,RSAD2,SOCS1,STAT1 | 26 | 21 | Replication of RNA virus |
| ACKR2 | IFIT2,IRF7,STAT1 | 24 | 20 | Cell movement |
| ACKR2 | IRF7,RSAD2,STAT1 | 23 | 19 | Replication of RNA virus |
| DDX58 | IFI35,RSAD2,STAT1 | 23 | 19 | Infection of cells |
| XBP1 | APOA1,APP,ESR1 | 22 | 19 | Binding of lipid |

**Table S5**. Regulatory networks activated in the liver of male fathead minnow in response to exposure to Mixture of three chemicals. The Regulatory network was generated using Ingenuity Pathways Analysis (IPA) software.

| **Regulators** | **Target Molecules in Dataset** | **Diseases & Functions** |
| --- | --- | --- |
| FOXC2,  histone deacetylase,PPARÎ±-RXRÎ± | ACAA1,ACOX1,APOA1,CXCR4,CYP24A1,ID2,INSR,JUNB,LPL,NEUROD1,PDGFRB,PPARGC1A | Cell viability of tumor cell lines,  Cleavage of lipid,  Movement Disorders,  Organismal death,  Oxidation of fatty acid,  Transport of molecule |
| EDN1,FOXO1,IKBKG,IL15,IL2,IL21,KITLG,LEP,MAPK14,NFKBIA,SOCS3,TLR2,TLR3,TLR4,TNFSF11,TSH | ACADM,ADRA1A,APOA1,APP,ATF3,BCL6,BIRC5,C3,CLU,CXCR4,EGR1,ENPP2,EPHX2,ESR1,GADD45A,HBEGF,HSP90B1,ID2,IL7R,INSR,JUNB,LEMD3,MB,ODC1,PLAUR,PNPLA2,POR,PPARGC1A,SST,STAT1,TNFRSF1A,TPT1,XBP1 | Development of cardiovascular system |
| GNRH1,NELFB,NR5A2 | APOA1,APP,ATF3,CYP7A1,EGR1,FASN,FOS,GADD45A,GADD45B,INHA,JUNB,MAD2L1,NR1H4 | Cell viability,  Differentiation of cells,  G1/S phase transition,  Survival of organism,  Uptake of lipid |
| CEBPA,CREBBP,EIF2AK2,FOXO3,GNAS,IFNL1,IL6ST,NEDD9,P38 MAPK,POMC,PRKAA2,TGFB2 | ABCB1,ANXA1,APOA4,APOB,APP,ATF3,B2M,BHMT,BIRC5,C3,CCR7,CXCR4,CYP24A1,CYP27B1,CYP7A1,DGAT2,DSPP,EGR1,EIF2AK3,ESR1,EVPL,FABP1,FASN,FLNA,FOS,G6PC,GADD45A,GAPDH,GSTP1,HBEGF,HMGCS2,HPX,HSPA5,HSPG2,ID2,IFIT2,IGFBP1,IL6R,INHA,INSR,JUNB,LEMD3,LPL,MDM2,NEUROD1,NR1H4,PIM2,PLAUR,PLIN2,PPARGC1A,PPL,SCD,SERPINE2,SGK1,SOCS1,SOCS3,SST,STAT1,TDRD7,TF,TNFRSF1A,UBE2C | Degradation of lipid,  Focal necrosis of liver,  Gastrointestinal neoplasia,  Inflammation of liver,  Movement Disorders,  Size of body |
| Ifn,Ifnar,IRF1,IRF3 | B2M,C3,CFH,IFIT2,IRF7 | Morbidity or mortality |
| F7,HOXA5,KIAA1524,PPARÎ±-RXRÎ±,SCP2 | ACAA1,ACOX1,APOA1,EGR1,FABP1,FOS,GADD45A,GADD45B,HBEGF,LPL,MDM2,PDGFRB,PLAUR,SAT1 | Cell viability,oxidation of fatty acid |
| EDN1,IKBKG,IL2,IL21,KITLG,LEP,MAPK14,TLR2,TLR3,TNFSF11,TSH | ACADM,ADRA1A,APOA1,APP,ARG2,ATF3,ATP5J2,B2M,BCL6,BIRC5,C3,CCR7,CLU,CTSL,CXCR4,CYP24A1,EGR1,ENPP2,EPHX2,ESR1,FASN,FOS,GADD45A,HSP90B1,ID2,IL7R,INSR,JUNB,MBP,MCL1,MDM2,ODC1,PLAUR,PLIN2,PNP,PNPLA2,POR,PPARGC1A,SCD,SGK1,SOCS1,SOCS3,SST,STAT1,TNFRSF1A,TPT1,TSPAN5,XBP1 | Development of cardiovascular system,  Urological cancer |
| CEBPA,CREBBP,TGFB2 | ARG2,C3,CXCR4,CYP24A1,DGAT2,EGR1,FOS,GSTP1,HBEGF,HSPG2,ID2,INHA,INSR,LPL,MDM2,NEUROD1,NR1H4,PLIN2,PPARGC1A,SCD,SERPINB1,SOCS3,TNFRSF1A | Fibrosis,  Size of body |
| IFNG,NELFB,P38 MAPK,PDGF BB,PIM1 | ATF3,BIRC5,C3,GADD45A,GADD45B,GHR,HRAS,ID2,INSR,MDM2,NR1H4,STAT1,TNFRSF1A | Focal necrosis of liver,  G1/S phase transition |
| ACKR2,HMGA1,Ifnar,NR5A2 | APOA1,C3,ESR1,FOS,INHA,INSR,IRF7,JUNB,NR1H4,PIM2,RSAD2,STAT1 | Survival of organism |
| CEBPB,GCG,ZBTB20 | ACADM,G6PC,GCK,IGFBP1,PPARGC1A,SCD | Gluconeogenesis |
| ERBB2,RELA,SMAD3 | EGR1,JUNB,MDM2,TF | Binding of synthetic promoter |
| BCR (complex),FGF2,HGF,ZBTB20 | ANG,AQP3,ATF3,BCL6,BIRC5,CASP2,CCR7,CTSL,CXCR4,DSPP,EGR1,ENPP2,FASN,FKBP5,FLNA,FOS,G6PC,GADD45A,GHR,HSP90B1,HSPA5,IGFALS,IGFBP1,IGFBP2,JUNB,KMT2A,KRT17,KRT18,LRP8,MAD2L1,MBP,MDM2,ODC1,PDGFRB,PLAUR,PNRC1,PPAP2B,PPARGC1A,PTTG1,SCD,SGK1,SOCS3,TF,TFPI,TNFRSF1A,UBE2C,XBP1 | Gastrointestinal neoplasia,  Movement Disorders,  Synthesis of protein |
| PI3K (complex),TLR3,Vegf | ABCB1,ATF3,BIRC5,C3,CLU,CXCR4,EGR1,ENPP2,FKBP5,FLNA,FOS,FRY,GSTP1,IFIT2,IGFBP1,IL7R,INHA,KMT2A,LIPA,LIPG,LRP8,MAD2L1,MAOB,PC,PLAUR,PPAP2B,SGK1,SLC6A3,SOCS3,STAT1,TNFRSF1A,TRIM25,UBE2C | Cleavage of lipid,  Gastrointestinal neoplasia,  Inflammation of liver |
| HGF,IL4,LEP,MYC,TGFB1 | ACADM,ACOX1,ACSL3,ACSS1,ALDOB,ANKH,APOA1,APOA4,APOB,APOE,APP,ARG2,ATF3,B2M,B4GALNT1,BCL6,BIRC5,C1S,C3,CASP2,CCR7,CD163L1,CD209,CENPE,CLU,CTSL,CXCR4,CYP24A1,CYP2F1,CYP7A1,EGR1,EIF4E,EPHX2,ESR1,FABP2,FASN,FHL1,FLNA,FOS,G6PC,GADD45A,GAPDH,GPR21,HNRNPH1,HSP90AA1,HSPA5,HSPG2,IGFBP2,IL17RC,IL7R,JUNB,KRT18,KRT8,LEMD3,LIPA,LPL,LRP8,MBP,MCL1,MDM2,METAP1,MFAP4,MSMB,MTHFD2,NOP56,ODC1,OSR2,PDGFRB,PDXK,PEX19,PLAUR,PLIN2,PNP,POLA1,PPARGC1A,PPL,PRC1,PTTG1,RPL13,RPS12,RPS18,SAT1,SCD,SCP2,SERPINA1,SERPINB1,SERPINF2,SGK1,SLC7A2,SOCS1,SOCS2,SOCS3,SPC25,SST,STAT1,STUB1,THY1,TNFRSF1A,TUBB4B,UBE2C,UCK2,UGT1A1,XBP1,ZWINT | Mass of epididymal fat,  Movement Disorders,  Pelvic cancer,  Urogenital cancer |
| ERBB2,FOXO1,FOXO3,MYC,SMAD4 | ACAA2,ADARB1,AIFM2,ALDOB,APOA1,APOA4,APOB,APP,BCL6,BIRC5,CASP2,CLU,CYP7A1,DBI,DRC7,EGR1,EIF4E,ELL2,EPHX2,ESR1,ETV5,EVPL,FABP1,FASN,FLNA,FOS,G6PC,GADD45A,GAPDH,GCK,HMGCS2,HSP90AA1,HSPA5,IL7R,INSR,JUNB,LEMD3,LPL,MB,MBP,MDM2,ME2,MTHFD1,NOP56,NUSAP1,ODC1,PDGFRB,PDHA1,PDIA4,PLAUR,PMEPA1,POR,PPARGC1A,PPP1R15A,PRC1,RPL13,RPL9,SAT1,SCD,SERPINA1,SGK1,TDO2,TF,THY1,TNFRSF1A,UBE2C,UGT1A1,XBP1 | Liver tumor,  Movement Disorders |
| ZAP70 | ANG,ANXA1,CCR7,CD69,HSP90AA1,SCD | Differentiation of cells |
| SMAD4 | APOA1,APOA4,APOB,FOS,GADD45A,LEMD3,MDM2,PDGFRB,POR,SCD,SGK1 | Liver tumor,  Transport of molecule |
| CSF2,CSF3,IFNAR1,IL1B,IL21,Interferon alpha,MAPK14,NFkB (complex),OSM,SOCS1,STAT3,Tlr,TNF,Vegf | ABCB1,ACADM,ACOX1,ACSL3,ANXA1,APOA1,APOB,APOE,APP,ARG2,ATF3,ATP2B1,ATP5J2,B2M,BCL6,BIRC5,C1S,C3,CASP2,CCR7,CD163L1,CD209,CENPE,CFH,CHD1,CLU,CTSL,CXCR4,CYP7A1,DPYS,EIF4E,ELL2,ENPP2,EP300,ESR1,FASN,FGA,FGG,FLNA,FOS,G6PC,GSTP1,GZMK,HSPG2,IGFBP1,IGFBP2,IL17RC,IL7R,INSR,JUNB,KIF20A,KMO,LRP8,MAOB,MBP,MCL1,MDM2,NEUROD1,ODC1,P2RX7,PCP4,PDAP1,PLAUR,PLIN2,PPP1R15A,PRC1,SAA2,SAT1,SCD,SERPINA1,SERPINB1,SERPING1,SGK1,SOCS2,SOCS3,SPC25,STAT1,TFPI,UBE2C,UGT1A1 | Pelvic cancer,  Urogenital cancer |
| Growth hormone,IL5 | BCL6,EGR1,FOS,G6PC,GADD45A,HIGD1A,HSPA5,IGFALS,LPL,SGK1,THY1,UCK2,XBP1 | Movement disorders |
| IL1B | APOB,APOE,ATF3,C3,CCR7,CXCR4,CYP7A1,EIF4E,ENPP2,EP300,ESR1,FOS,HSPG2,IGFBP1,INSR,MCL1,NEUROD1,NR1H4,ODC1,SOCS1,SOCS3,STAT1 | Degradation of lipid,  Urological cancer |
| SLC13A1 | ACSL5,COPB2,HSPA5,IGFBP2,XBP1 | Cell viability |
| TLR9 | ATF3,CCR7,CISH,NR1H4,SOCS1 | Inflammation of body cavity |
| HNF4A | ABCB11,ABHD6,ACOX1,APOA1,APOB,APOE,BCL6,C3,EGR1,GAPDH,HPX,HSPA5,IGFBP1,KRT8,LIPA,NR1H4,SCD,SCP2,STAT1 | Inflammation of body cavity |
| CEBPB | APOB,BHMT,C3,DGAT2,DSPP,FHL1,FOS,G6PC,HPX,HSP90AA1,INSR,LEMD3,MBP,PLAUR,PLG,PPP1R15A,SAA2,SCD,SERPINA1,SGK1,SLC38A2,SOCS3,SYCP1,TF | Liver cancer |
| IFN alpha/beta | IRF7,RSAD2,SOCS1,STAT1 | Survival of organism |
| SASH1 | IRF7,RSAD2,SOCS1,STAT1 | Survival of organism |
| NFATC2 | IFIT2,IRF7,MDM2,SOCS3 | Morbidity or mortality |
| DOCK8 | IRF7,RSAD2,SOCS1,STAT1 | Survival of organism |
| IL12 (complex) | CCR7,CISH,SOCS1,TNFRSF1A | Inflammation of body cavity |
| IFNA2 | IL10RB,SOCS1,STAT1,TNFRSF1A | Inflammation of liver |
| SAMSN1 | HBEGF,SOCS3,STAT1,XBP1 | Development of cardiovascular system |
| MAVS | IFIT2,IRF7,SOCS3 | Morbidity or mortality |
| TICAM1 | IFIT2,IRF7,SOCS3 | Morbidity or mortality |
| DDX58 | IFIT2,IRF7,SOCS3 | Morbidity or mortality |
| LIF | ACAA2,KNG1,LPL | Cleavage of lipid |
| IFN Beta | CCR7,SOCS1,STAT1 | Inflammation of liver |
| IFNA1/IFNA13 | CXCL11,SOCS1,STAT1 | Cell viability |
| IL6 | ABCB11,ACOX1,ANXA1,APOA1,APOB,APOE,APP,ATF3,BCL6,BIRC5,C3,CCR7,CD163L1,CD209,CDKN2D,CENPE,CES2,CISH,CLU,CXCR4,DVL1,EGR1,ENPP2,FGA,FGB,FGG,FLI1,FOS,G6PC,GADD45A,GALE,HPX,HSPA5,ID2,IGFBP1,IL17RC,IL6R,IL7R,ITLN1,JUNB,KRT18,KRT8,LIG1,LPL,MAD2L1,MCL1,NR1H4,PLG,PPP1R15A,PPRC1,PTTG1,SAA2,SERPINA1,SGK1,SOCS1,SOCS2,SOCS3,SPC25,STAT1,TF,UBE2C,XBP1 | Abdominal neoplasm |
| HNF4A | C3,FABP1,FABP2,SCD,SCP2 | Incorporation of long chain fatty acid |
| IRF7 | MCL1,RSAD2,SOCS1,STAT1 | Survival of organism |
| TRIM24 | CYP24A1,SOCS1,SOCS2,STAT1 | Size of body |
| IL1B,TNF | ESR1,NR1H4,SOCS1 | Degeneration of liver |

**Table S6.** The predicted directional effects on diseases and biological functions that are overrepresented in the dataset in the male fathead minnow liver in response to NP, BPA, DEHP and mixture exposure. These regulators are predicted based on the significant overrepresented genes in the dataset using Ingenuity Pathways Analysis (IPA) software. (The Z-scores >2 or < -2 are considered significant).

| **Diseases and Bio Functions** | **Z-Score** | | | |
| --- | --- | --- | --- | --- |
|  | **NP** | **BPA** | **DEHP** | **Mix** |
| Organismal death | 0.774 | -1.132 | 4.892 | 3.982 |
| Abdominal neoplasm | -0.595 | -0.128 | 1.375 | 3.268 |
| Digestive organ tumor | -1.111 | -0.268 | 0.853 | 3.108 |
| Abdominal cancer | -0.289 | 0.257 | 1.028 | 2.79 |
| Binding of synthetic promoter | 0.902 | 1.551 | 0.587 | 2.768 |
| Inflammation of liver | -0.555 | -0.18 | 2.895 | 2.747 |
| Digestive tract cancer | -0.649 | 0.292 | 0.79 | 2.448 |
| Liver tumor | -0.305 | 0.559 | 1.228 | 2.385 |
| Movement disorders | 1.928 | 0.676 | 2.278 | 2.309 |
| Urogenital cancer | 0.378 | 0 | 1.342 | 2.236 |
| Pelvic cancer | 0.378 | 0 | 1.342 | 2.236 |
| Urological cancer | 0.378 | 0 | 1.342 | 2.236 |
| G1/S phase transition | 0.82 | 0.049 | 0.306 | 2.233 |
| Gastrointestinal neoplasia | -0.943 | -0.492 | 0.314 | 2.213 |
| Morbidity or mortality | -0.343 | 0.808 | 1.522 | 2.183 |
| Hepatocellular carcinoma | -0.855 | -0.301 | 1.258 | 2.18 |
| Focal necrosis of liver | -1.526 | -0.388 | 2.158 | 2.158 |
| Synthesis of protein | -1.033 | -0.111 | -1.396 | 2.097 |
| Fibrosis | 0 | 0 | 1.985 | 2.065 |
| Liver cancer | -0.332 | 0.676 | 0.866 | 2.062 |
| Inflammation of body cavity | -1.382 | -1.142 | 2.068 | 2.031 |
| Mass of liver | -2.082 | -2.354 | 1.605 | 1.949 |
| Cell death of liver | 0.123 | -0.204 | 3.417 | 1.682 |
| Necrosis of liver | 0.123 | -0.204 | 3.417 | 1.682 |
| Apoptosis of liver | 0.279 | -0.569 | 2.136 | 1.257 |
| Apoptosis of liver cells | 0.279 | -0.569 | 2.136 | 1.257 |
| Feeding | -2.573 | 0 | -0.616 | 1.197 |
| Morphology of body cavity | 1.196 | -0.42 | 2.508 | 1.196 |
| Cell death of liver cells | 0.559 | -0.189 | 2.611 | 1.035 |
| Ingestion by rodents | -2.84 | -0.747 | -0.459 | 0.852 |
| Cell death | 1.623 | -0.199 | 3.068 | 0.785 |
| Cell death of tumor cell lines | 1.295 | 0.36 | 2.515 | 0.687 |
| Ingestion by mice | -2.673 | -0.482 | -0.327 | 0.6 |
| Metabolism of amino acids | 1.4 | 2.414 | -1.4 | 0.6 |
| Morphology of digestive system | 0.416 | 2.408 | 1.423 | 0.579 |
| Apoptosis of tumor cell lines | 1.687 | 0.935 | 2.319 | 0.494 |
| Replication of RNA virus | -0.509 | 1.311 | -2.197 | 0.371 |
| Necrosis | 1.43 | -0.514 | 3.193 | 0.359 |
| Quantity of blood cells | -1.894 | -1.609 | -2.256 | 0.248 |
| Binding of cells | 0.948 | 4.297 | -0.253 | 0.239 |
| Accumulation of lipid | -2.71 | -0.987 | -0.778 | 0.221 |
| Replication of virus | -0.941 | 1.268 | -2.147 | 0.202 |
| Binding of lipid | 1.964 | 1.949 | 2.152 | 0.186 |
| Apoptosis | 1.183 | -0.254 | 3.161 | 0.058 |
| Quantity of cells | -1.14 | -0.604 | -2.634 | -0.016 |
| Storage of lipid | -0.911 | 0.045 | -2.395 | -0.025 |
| Viral infection | -0.525 | 2.612 | -2.97 | -0.027 |
| Quantity of leukocytes | -2.197 | -1.437 | -2.496 | -0.11 |
| Hepatic steatosis | -2.311 | -0.766 | 0.221 | -0.131 |
| Arthropathy | 2.683 | 1.8 | 0.447 | -0.248 |
| Steroid metabolism | 2.128 | 0.794 | 0.377 | -0.285 |
| Secretion of cholesterol | -0.555 | -1.067 | -2.393 | -0.342 |
| Arthritis | 2.799 | 1.479 | 0.191 | -0.494 |
| Apoptosis of prostate cancer cell lines | 1.987 | -0.437 | 2.395 | -0.509 |
| Fatty acid metabolism | 0.366 | 0.792 | -2.413 | -0.511 |
| Concentration of fatty acid | -2.332 | -1.065 | -0.79 | -0.615 |
| Synthesis of lipid | -0.714 | 0.517 | -2.199 | -0.685 |
| Rheumatic disease | 2.73 | 1.814 | 0.806 | -0.725 |
| Cell movement of tumor cell lines | 0.239 | -0.86 | -2.313 | -0.727 |
| Efflux of cholesterol | 1.537 | 0.645 | -0.652 | -0.727 |
| Release of lipid | 0 | 0 | -2.132 | -0.733 |
| Infection by RNA virus | -0.612 | 2.571 | -2.562 | -0.775 |
| Weight gain | -2.045 | -0.184 | -1.394 | -0.825 |
| Infection of cells | -0.619 | 2.556 | -2.563 | -0.837 |
| Cell movement | -0.125 | 0.626 | -2.066 | -0.858 |
| Quantity of insulin in blood | -0.929 | 0 | -2.128 | -0.88 |
| Cellular homeostasis | -1.829 | -0.533 | -3.347 | -0.956 |
| Flux of lipid | 2.179 | 1.235 | -0.534 | -0.979 |
| Proliferation of breast cancer cell lines | -0.075 | 0.2 | -2.85 | -1.051 |
| Synthesis of triacylglycerol | -1.255 | 1.241 | -2.09 | -1.15 |
| Efflux of phospholipid | 2.38 | 0 | -0.479 | -1.154 |
| Transport of lipid | 2.563 | 1.556 | -1.511 | -1.218 |
| Metabolism of triacylglycerol | -1.402 | 1.228 | -2.188 | -1.304 |
| Secretion of molecule | 0.937 | -1.519 | -2.546 | -1.528 |
| Proliferation of tumor cell lines | -0.812 | -0.386 | -3.059 | -1.575 |
| Metabolism of nucleic acid component or derivative | 1.393 | 2.02 | -1.982 | -1.745 |
| Metabolism of nucleotide | 1.393 | 2.02 | -1.982 | -1.745 |
| Oxidation of lipid | -0.261 | 1.517 | -2.114 | -1.933 |
| Degradation of lipid | 1.037 | 0.688 | -0.01 | -2.176 |
| Gluconeogenesis | -0.6 | 0 | 0 | -2.2 |
| Incorporation of long chain fatty acid | 0 | 0 | 0 | -2.213 |
| Size of body | 1.356 | 0.719 | -1.869 | -2.221 |
| Cell survival | -1.651 | 0.016 | -4.181 | -2.223 |
| Survival of organism | -0.765 | 0.114 | -1.583 | -2.274 |
| Mass of epididymal fat | 0.632 | 0.707 | -1.265 | -2.333 |
| Differentiation of cells | 0.552 | 1.271 | -2.165 | -2.551 |
| Oxidation of fatty acid | -0.061 | 2.02 | -1.824 | -2.571 |
| Transport of molecule | 1.434 | 0.889 | -3.208 | -2.651 |
| Cell viability | -1.469 | 0.22 | -3.975 | -2.84 |
| Uptake of lipid | -0.185 | 1.183 | -2.095 | -2.966 |
| Cleavage of lipid | 0.918 | 1.875 | -1.109 | -3.014 |
| Cell viability of tumor cell lines | -2.368 | -0.279 | -4.545 | -3.096 |
| Development of cardiovascular system | -0.257 | 0 | -1.484 | -3.1 |
| Apoptosis of blood cells | 1.074 | 0 | 2.297 | 0 |
| Transformation of fibroblast cell lines | 0.679 | 0.752 | -2.154 | 0 |
| Quantity of lymphocytes | 0 | 0 | -2.984 | 0 |
| Quantity of mononuclear leukocytes | -1.611 | 0 | -3.28 | 0 |
| Conversion of fatty acid | 0.579 | 2.607 | 0 | 0 |
| Infection of kidney cell lines | 0 | 2.54 | 0 | 0 |
| Conversion of acyl-coenzyme A | 0 | 2 | 0 | 0 |
| Binding of colon cancer cell lines | 0 | 2 | 0 | 0 |
| Synthesis of amino acids | 0 | 2 | 0 | 0 |
| Uptake of monosaccharide | 2.029 | -0.17 | 0 | 0 |
